# Supplementary figures and images for: Developmental outcomes of an individualised complementary feeding intervention for stunted children: a substudy from a larger randomised controlled trial in Guatemala
Source: BMJ Paediatr Open. 2018 Oct 3;2(1):e000314. doi: 10.1136/bmjpo-2018-000314 (PMC6173251; doi:10.1136/bmjpo-2018-000314)

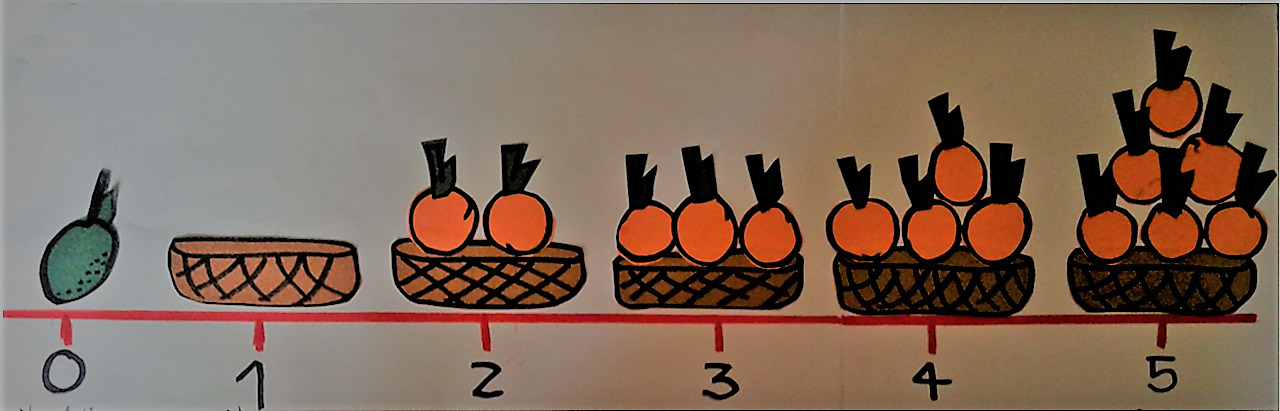

Supplement: Supplementary file 1 [file bmjpo-2018-000314supp001.jpg]
