# Supplementary material for: Developmental outcomes of an individualised complementary feeding intervention for stunted children: a substudy from a larger randomised controlled trial in Guatemala
Source: BMJ Paediatr Open. 2018 Oct 3;2(1):e000314. doi: 10.1136/bmjpo-2018-000314 (PMC6173251; doi:10.1136/bmjpo-2018-000314)
Supplement: Supplementary file 2 [file bmjpo-2018-000314supp002.docx]

**Supplementary Table 1: Baseline Characteristics of Participants who Completed the Study vs Lost to Follow-up or Unable to Complete BSID-III Data Collection**

| **Characteristics^1^** | **Completed Study (N=147)** | **Lost to follow up or Incomplete BSID-III (N=63)** | **P value^2^** |
| --- | --- | --- | --- |
| **Maternal Characteristics**  Age – yrs  Education – yrs  Literacy – no. (%)  Parity | 27.07 ± 6.81  2 [0 – 4]  72 (50)  3 [2 – 5] | 26.54 ± 6.41  2 [0 – 3]  34 (54)  3 [2 – 4] | 0.60  0.90  0.51  0.62 |
| **Child Characteristics**  Male – no. (%)  Age at BSID III evaluation – months^3^ Height-for-age Z-score  Weight-for-age Z-score  Weight-for-length Z-score | 89 (61)  16.27 ± 5.18  -3.37 ± 0.66  -1.95 ± 0.77  -0.20 ± 0.91 | 37 (59)  15.00 ± 4.34  -3.49 ± 0.87  -2.02 ± 0.79  -0.23 ± 0.76 | 0.81  0.22  0.31  0.52  0.80 |
| **Feeding Practices Indicators**  Minimum dietary diversity – no. (%)  Minimum meal frequency – no. (%)  Minimum acceptable diet – no. (%) | 88 (60)  122 (83)  74 (50) | 42 (67)  58 (92)  41 (62) | 0.35  0.13  0.05 |
| **Household Characteristics**  Family Poverty Score  Family Care Indicators Score | 27.23 ± 10.08  8.54 ± 2.33 | 27.65 ± 11.52  8.49 ± 2.46 | 0.79  0.90 |
| **BSID-III Subscales Z-scores**^3^  Cognitive  Receptive Language  Expressive Language  Fine Motor  Gross Motor  Socioemotional | 0.00 ± 1.00  -0.01 ± 0.96  -0.01 ± 0.96  -0.01 ± 1.01  0.03 ± 0.98  0.06 ± 1.05 | -0.02 ± 0.89  0.06 ± 1.12  0.04 ± 1.09  0.04 ± 0.83  -0.18 ± 0.98  -0.25 ± 1.03 | 0.93  0.73  0.83  0.81  0.31  0.15 |

^1^ Plus minus values are means ± SD. ^2^P values calculated using Student’s t-test or Wilcoxon Mann-Whitney text for continuous variables, and the Chi2 or Fischer’s exact test for categorical variables, as appropriate. ^3^ Participants with BSID-III at study entry who were lost to follow up are included in the third column (n=28).
