# Supplementary material for: Developmental outcomes of an individualised complementary feeding intervention for stunted children: a substudy from a larger randomised controlled trial in Guatemala
Source: BMJ Paediatr Open. 2018 Oct 3;2(1):e000314. doi: 10.1136/bmjpo-2018-000314 (PMC6173251; doi:10.1136/bmjpo-2018-000314)
Supplement: Supplementary file 3 [file bmjpo-2018-000314supp003.docx]

**Supplementary Table 2: Baseline Characteristics of Participants Included versus Excluded in Outcome Analysis**

| **Characteristics^1^** | **Included**  **(N=100)** | **Excluded**  **(N=47)** | **P value^2^** |
| --- | --- | --- | --- |
| **Maternal Characteristics**  Age – yrs  Education – yrs  Literacy – no. (%)  Parity | 27.14 ± 6.61  2 [0 – 4]  54 (54)  3 [2 – 5] | 26.94 ± 7.28  2 [0 – 4]  18 (38)  2 [1 – 5] | 0.87  0.20  0.08  0.16 |
| **Child Characteristics**  Male – no. (%)  Age at BSID III evaluation – months Height-for-age Z-score  Weight-for-age Z-score  Weight-for-length Z-score | 59 (59)  13.60 ± 3.95  -3.33 ± 0.71  -1.91 ± 0.79  -0.10 ± 0.93 | 30 (64)  21.97 ± 1.71  -3.47 ± 0.55  -2.04 ± 0.73  -0.41 ± 0.83 | 0.58  0.0000  0.22  0.32  0.05 |
| **Feeding Practices Indicators**  Minimum dietary diversity – no. (%)  Minimum meal frequency – no. (%)  Minimum acceptable diet – no. (%) | 54 (54)  88 (88)  49 (49) | 34 (72)  34 (72)  25 (53) | 0.05  0.03  0.64 |
| **Household Characteristics**  Family Poverty Score  Family Care Indicators Score | 27.12 ± 10.61  8.47 ± 2.41 | 27.47 ± 8.95  8.68 ± 2.15 | 0.85  0.61 |

^1^ Plus minus values are means ± SD. ^2^P values calculated using Student’s t-test or Wilcoxon Mann-Whitney text for continuous variables, and the Chi2 or Fischer’s exact test for categorical variables, as appropriate.
