# Supplementary material for: Developmental outcomes of an individualised complementary feeding intervention for stunted children: a substudy from a larger randomised controlled trial in Guatemala
Source: BMJ Paediatr Open. 2018 Oct 3;2(1):e000314. doi: 10.1136/bmjpo-2018-000314 (PMC6173251; doi:10.1136/bmjpo-2018-000314)
Supplement: Author's manuscript [file bmjpo-2018-000314.draft_revisions.pdf]

BMJ Paediatrics Open

BMJ Paediatrics Open is committed to open peer review. As part of this commitment we make the peer review history of every article we publish publicly available.

When an article is published we post the peer reviewers' comments and the authors' responses online. We also post the versions of the paper that were used during peer review. These are the versions that the peer review comments apply to.

The versions of the paper that follow are the versions that were submitted during the peer review process. They are not the versions of record or the final published versions. They should not be cited or distributed as the published version of this manuscript.

BMJ Paediatrics Open is an open access journal and the full, final, typeset and author-corrected version of record of the manuscript is available on our site with no access controls, subscription charges or pay-per-view fees (http://bmjpaedsopen.bmj.com).

If you have any questions on BMJ Paediatrics Open's open peer review process please email info.bmjpo@bmj.com

BMJ Paediatrics Open

Confidential: For Review Only

Developmental outcomes of an individualized complementary feeding intervention for stunted children: A sub-study from a larger randomized controlled trial in Guatemala

Journal:	*BMJ Paediatrics Open*
Manuscript ID	bmjpo-2018-000314
Article Type:	Original article
Date Submitted by the Author:	21-May-2018
Complete List of Authors:	Martinez, Boris; Wuqu' Kawoq,<br>Cardona, Sayra; Universidad del Valle de Guatemala<br>Rodas, Patricia; Universidad del Valle de Guatemala<br>Lubina, Meri; Universidad del Valle de Guatemala<br>Gonzalez, Ana; Universidad del Valle de Guatemala<br>Farley Webb, Meghan; Wuqu' Kawoq,<br>Grazioso, Maria del Pilar; Universidad del Valle de Guatemala<br>Rohloff, Peter
Keywords:	Child Psychology, General Paediatrics, Nutrition, Tropical Paediatrics

SCHOLARONE™
Manuscripts

TITLE PAGE

Developmental outcomes of an individualized complementary feeding intervention for stunted children: A sub-study from a larger randomized controlled trial in Guatemala.

Boris Martinez MD[1], Sayra Cardona MA[2], Patricia Rodas MSc[2], Meri Lubina MA[2], Ana Gonzalez MA[2], Meghan Farley Webb PhD[1,3], Maria del Pilar Grazioso PhD[2], Peter Rohloff MD PhD[1,4,5]

[1]Wuqu' Kawoq | Maya Health Alliance, Santiago Sacatepéquez, Guatemala

[2]Department of Psychology, Universidad del Valle de Guatemala, Guatemala City, Guatemala

[3]Department of Anthropology & Sociology, Albion College, Albion, MI

[4]Division of Global Health Equity, Brigham and Women's Hospital, Boston, MA

[5]Corresponding Author. Peter Rohloff, Brigham and Women's Hospital, 75 Francis Street, Boston, MA, 02115. Phone: 617-278-0055. Fax: 888-372-2354. Email: prohloff@bwh.harvard.edu

Word count: 2538

Confidential: For Review Only

KEY WORDS:

Stunting, early child development, randomized clinical trial, Guatemala, indigenous, community health workers

Confidential: For Review Only

WHAT IS KNOWN ABOUT THIS SUBJECT

- Stunting is the most common cause of early childhood developmental delays, and nearly half of Guatemalan children are stunted, with higher prevalence among the Guatemalan Maya-indigenous population.
- Stunting early in life has deleterious long-term impacts on educational attainment, intellectual outcomes and adult economic earning potential. Few investigations of these effects exist for the Guatemalan indigenous Maya population.
- Complementary feeding practices interventions are the cornerstone for stunting prevention and treatment, however investigation of the effects of these interventions on developmental outcomes are limited.

WHAT THIS STUDY ADDS

- Both standard and augmented behavior change approaches to improved complementary feeding practices by caregivers have important positive impacts on the development of stunted children.
- Current public malnutrition reduction strategies being implemented in indigenous Maya communities in Guatemala can improve childhood developmental outcomes.
- Developmental assessments can be feasibly adapted and contextualized to a rural, indigenous population at risk.

Confidential: For Review Only

STRUCTURED ABSTRACT

Objective: Stunting is one of the most common causes of early child developmental delay worldwide; Guatemala has the fourth highest rate of stunting globally. The goal of this study was to examine the impact of an intensive community health worker-led complementary feeding caregiver education intervention on early child development in Guatemala. We hypothesized that the intervention would improve child development over usual care.

Design: A sub-study from a larger individually randomized (1:1 allocation ratio), parallel-group superiority trial, with blinding of study staff collecting outcomes data.

Setting: Rural, indigenous Maya communities in Guatemala.

Participants: 210 stunted children (height-for-age Z score less than or equal to -2.5) 6-24 months old and their caregivers, previously randomized to usual care (106) or an intensive complementary feeding intervention (104) were invited to participate in the developmental evaluation. 84 dyads in the intervention and 91 in the usual care arm agreed to participate and provided informed consent.

Interventions: Community health workers conducted monthly home visits for 6 months, providing usual care or usual care plus individualized caregiver complementary feeding education.

Main Outcome Measures: The primary outcomes were change in Z-scores for the fine and gross motor, receptive and expressive language, cognitive, and socioemotional subscales of the Bayley Scales of Infant Development (BSID), Third Edition.

Results: 100 individuals were included in the final analysis, 47 in the intervention and 53 in the usual care arm. No statistically significant differences in age-adjusted scores between the intervention and usual care arms were observed for any developmental subscale. However,

Confidential: For Review Only

meaningful improvements within-subjects in age-adjusted scores in both arms were observed from study enrollment to completion (median duration between measurements was 189 days [IQR 182 – 189]. Average change for developmental subscales were 0.45 (95% CI 0.23 – 0.67) Z-scores in the intervention arm, and 0.43 (95% CI 0.25 – 0.61) in the usual care arm.

Conclusions: An intensive complementary feeding intervention did not significantly improve developmental outcomes more than usual care in a population of stunted, indigenous Guatemalan children. However, both the intensive and usual care interventions had significant positive impacts on developmental outcomes.

Trial registration: Clinicaltrials.gov NCT02509936

Funding: Grand Challenges Canada grant SB-1726251050

Stage: Results

Confidential: For Review Only

INTRODUCTION

In low and middle-income countries, 43% of children under five are at risk of not reaching their developmental potential, and 70% of the attributable risk for delays in early childhood development (ECD) is due to stunting, or low height-for-age.[1, 2] Guatemala, a Central American country of 17 million inhabitants, has the highest rate of stunting in the Western Hemisphere.[3] The burden of stunting disproportionately affects the rural, indigenous Maya population, where stunting often exceeds 70% and complementary feeding and dietary diversity indicators are poor.[4-6]

Early scientific research in Guatemala was critical to the international community's understanding of stunting's impact on human capital. The Institute of Nutrition of Central America and Panama cohort study—conducted from 1969-1977, with long-term follow-up—demonstrated the deleterious impacts of stunting on educational attainment, intellectual outcomes, and adult economic earning potential.[7] Subsequently, there have been extensive investigations of stunting's nutritional and sociodemographic correlates in the indigenous Maya population. However, there have been few investigations of developmental outcomes or of the impact of contemporary nutritional interventions on ECD.[8-11]

Given the gaps in adequate complementary feeding practices and diet quality, interventions to promote caregiver knowledge around infant-child feeding are an important strategy in Guatemala.[5, 6, 12] This strategy is supported by international evidence, with several studies showing that linear growth is strongly correlated with better development and that complementary feeding education interventions improve stunting.[11, 13-15] However, there has been limited direct investigation of the effect of complementary feeding on developmental outcomes.[16-18] This is an important knowledge gap, since the political will for nutrition

Confidential: For Review Only

interventions in Guatemala is fragile, and more robust evidence of impact would have political significance.

In this study, we contribute by assessing developmental outcomes in indigenous Maya infants in rural Guatemala. We hypothesized that an individualized, intensive approach to caregiver complementary feeding education would improve outcomes over usual care. We individually randomized child-caregiver dyads (aged 6-24 months, height-for-age Z-score <-2.5) to 6 months of usual care, which included home-based growth monitoring and micronutrient and food supplementation, versus usual care augmented with individualized complementary feeding education, using monthly structured interviews and active goal-setting to promote incremental improvements.

METHODS

Study Context. This study was conducted at Maya Health Alliance (MHA), a primary healthcare organization working in rural Maya communities. At MHA, nutrition community health workers (CHW) provide home-based services to children aged 6-24 months with stunting.[19] The study was conducted in Tecpán, Chimaltenango (pop. 95,000), with a settlement of agricultural Kaqchikel Maya families living 25 kilometers from the town center. The study was conducted according to the principles of Declaration of Helsinki and approved by the Institutional Review Boards of the Universidad del Valle de Guatemala and Maya Health Alliance.

Trial Design. This was a planned sub-study on developmental outcomes within a larger, individually randomized, two-arm trial comparing individualized complementary feeding caregiver education to usual care (Clinicaltrials.gov Identifier: NCT02509936), described in detail elsewhere.[20] Briefly, eligibility criteria included: age 6-24 months of age and length-for-

Confidential: For Review Only

age Z score less than or equal to -2.5 standard deviations (SD) by World Health Organization (WHO) standards.[21] Exclusion criteria were acute malnutrition (weight-for-length Z score of <= -2 SD) or severe medical illness. Study interventions were delivered by two CHW teams, each consisting of 2 CHWs. One team provided usual care while the other provided the intervention. Child-caregiver dyads were recruited and written informed consent obtained by a study staff member not involved in the intervention. At enrollment, child's anthropometric and dietary feeding practices as well as household demographic and socioeconomic characteristics were obtained. Household developmental stimulation data was gathered at enrollment using the Family Care Indicators (FCI) interview.[22] All subjects recruited for the larger study during the first five months of the larger study (n=210, Figure 1) were invited to participate in the sub-study. Study outcomes (subscale scores on the Bayley Scales of Infant Development, Third Edition [BSID-III]) [23] were obtained at 0 and 6 months by trained study psychologists from the Universidad del Valle de Guatemala, supported by MHA interpreters (Maya Kaqchikel/Spanish). Both psychologists and interpreters were blinded to study allocation.

Study Interventions. The study duration was 6 months. As described elsewhere,[20] the usual care arm received a monthly home-visit regimen from a team of CHWs including growth monitoring, daily multiple micronutrient powder supplement (ferrous fumarate 12.5 mg, zinc gluconate 5 mg, retinol acetate 300 mcg, folic acid 160 mcg, and ascorbic acid 30 mg; Prodipa S.A., Guatemala City, Guatemala); and a biweekly food ration (beans 1000 g., eggs 20 units, and *Incaparina* 900 g. [a common corn and soy-based food supplement; Alimentos S.A., Guatemala City, Guatemala]). For the intervention arm, subjects received usual care and monthly individualized caregiver counseling focused on improving meal frequency and dietary diversity for the child,[24] from a separate CHW team.

Confidential: For Review Only

Study Outcomes and Measure. The primary outcome was a change in Z-score for subscales of the BSID-III, calculated as described below. In preparation for use of the BSID-III, our team conducted a review of prior studies of psychometric testing with Guatemalan children, with the majority using previous versions of the BSID.[9-11, 25] Despite its technical complexity, the BSID is widely-used as a gold-standard instrument worldwide.[26, 27] Additionally, our lead psychologist (MPG) had extensive prior experience successfully using the tool in Guatemala. For these reasons, the BSID-III was chosen.

In preparation for testing, our team (interpreters, linguists, psychologists, anthropologists, nurses and physicians) reviewed existing BSID-III English and Spanish materials.[23, 28] Modifications for idiomatic Guatemalan Spanish and translations into Kaqchikel Maya were produced through group discussion and audience testing with volunteer caregivers and health workers. Forward translation, back translation, harmonization and cognitive debriefing were used to ensure accuracy and cultural adaptation. A visual Likert scale assisted with administration of socioemotional scales (Supplementary Figure 1). A small number of vocabulary and visual stimulus items were substituted to be culturally or context appropriate (e.g., sedan car substituted for pick-up truck).

Psychologists (bilingual Spanish/English) participated in a one week-long training in rural data collection and multicultural competencies. Interpreters (Kaqchikel Maya/Spanish speaking) participated in a one-week training with active BSID-III practice sessions. Senior study psychologist (MPG) supervised BSID-III training and data collection procedures.

Sample Size and Randomization. For the primary outcome, we calculated that enrollment of 72 subjects per arm would detect a change of 0.5 SD on the BSID-III composite score, with an

Confidential: For Review Only

170 alpha of 0.05, power of 80%, and 15% lost to follow-up. Allocation was by simple randomization from a computer-generated list of random numbers.

172 *Statistical Analyses.* Descriptive statistics were calculated using Stata 13. Family poverty scores (possible score range: 0-100) were calculated as described.[29] Overall FCI scores were calculated as the sum of 18 item scores.[22] Differences between arms were assessed using the Student's t-test or Wilcoxon-Mann-Whitney test for continuous variables and the chi-square or Fisher's exact test for categorical variables. Analysis was by intention-to-treat, except where loss to follow up prevented collecting outcome data.

178 Raw scores for each BSID-III subscale (cognitive, receptive and expressive language, fine and gross motor, socioemotional) were calculated as the sum of assessment items performed. BSID-III norms do not exist for Guatemala, and there are concerns about the validity of applying BSID-III norms from a different population.[30, 31] Furthermore, after data collection for the study was completed, interim ethics board review requested that we not use external norms in our analysis plan. Therefore study-internal Z-scores for each BSID-III subscale were calculated based on the raw scores distribution of each age-band during baseline measurements, an approach used in other studies.[32, 33] These were then used to calculate individual subject Z-scores for both 0 and 6 month timepoints.

187 We conducted an adjusted exploratory analysis to investigate the effect of prespecified covariates on the primary outcomes, including maternal parity and education, gender, number of under-five children in the home, household socioeconomic status, and age and length-for-age at enrollment. We constructed a hierarchical linear model for BSID-III subscales (MIXED function, Stata 13). Non-significant covariates were removed from the model using serial

Confidential: For Review Only

likelihood ratio tests.[34] The best fit model for all BSID-III subscales was chosen using the Breusch-Pagan Langrange multiplier test.

RESULTS

Participants. Eligible participants were recruited in rolling fashion from August - December 2015. Final participants exited in July 2016. A total of 210 children were eligible for the sub-study (104 intervention arm, 106 usual care arm). 84 children in the intervention and 91 in the control arm received BSID-III evaluation at study entry. Baseline characteristics of participants in the two study arms were well balanced, except for children in the intervention arm having greater minimum dietary diversity (Table 1).

Table 1: Baseline Demographic, Clinical, and BSID-III Characteristics of Study Participants

Characteristic[1]	Individualized Education (Intervention) Arm (N=84)	Usual Care Arm (N =91)	p Value[2]
**Maternal Characteristics**			
Age – yrs	26.08 ± 6.84	27.15 ± 6.59	0.29
Education – yrs	2 [0 – 3.5]	2 [0 – 4]	0.93
Literacy – no. (%)	44 (51.65)	47 (52.38)	0.92
Parity	2.5 [1 – 5]	3 [2 – 5]	0.43
**Child Characteristics**			
Male – no. (%)	49 (58.33)	57 (62.64)	0.56
Age at BSID III evaluation – months	16.32 ± 4.93	15.84 ± 5.21	0.53
Height-for-age Z-score	-3.40 ± 0.69	-3.41 ± 0.73	0.91
Weight-for-age Z-score	-2.01 ± 0.74	-1.94 ± 0.82	0.54
Weight-for-length Z-score	-0.26 ± 0.86	-0.13 ± 0.90	0.35
**Feeding Practices Indicators**			
Minimum dietary diversity – no. (%)	59 (70.24)	49 (53.85)	0.03
Minimum meal frequency – no. (%)	68 (80.95)	81 (89.01)	0.13
Minimum acceptable diet – no. (%)	51 (60.71)	43 (47.25)	0.07
**Household Characteristics**			

Confidential: For Review Only

# Children under 5 years	2 [1 – 2]	2 [1 – 2]	0.18
Family Poverty Score	28.33 ± 11.64	27.35 ± 9.17	0.53
Family Care Indicators Score	8.59 ± 2.63	8.47 ± 2.15	0.74
**BSID-III Subscales Z-scores**[3]			
Cognitive	-0.05 ± 1.00	0.04 ± 0.97	0.53
Receptive Language	-0.02 ± 0.96	0.02 ± 1.00	0.81
Expressive Language	0.01 ± 0.94	-0.01 ± 1.02	0.88
Fine Motor	-0.15 ± 0.94	0.14 ± 1.00	0.06
Gross Motor	-0.10 ± 0.91	0.10 ± 1.04	0.18
Socioemotional	0.05 ± 1.15	-0.02 ± 0.95	0.65

[1] Data presented as means ± SD, median [IQR], or no. (%), as appropriate.
[2] Students t-test or Wilcoxon-Mann-Whitney test for continuous variables, chi2 or Fischer's exact test for categorical variables, as appropriate.

The cumulative incidence of loss to follow-up was 18% (n=15) in the intervention and 14% (n=13) in the usual care arm (Figure 1). One child in the control arm discontinued treatment. Furthermore, 22 children in the intervention arm and 25 in the control arm with BSID-III data at study exit were older than 25.5 months-old. Baseline Z-score data distribution was not available for this older age-band. Supplementary Table 1 compares the characteristics of subjects who completed the study and both BSID-III evaluations (n=147) to those lost to follow-up or who did not complete both assessments (n=63). There were no statistically significant differences for major characteristics. Supplementary Table 2 compares the baseline characteristics of subjects excluded from the outcomes analysis due to age at study exit versus those included. Subjects excluded were similar to those included, except for older age and lower minimum meal frequency.

Outcomes. The analysis of primary outcomes was by intention-to-treat, including one child who discontinued treatment. 100 subjects were included in the primary analysis (47 intervention arm, 53 usual care arm; Figure 1). For developmental outcomes (Table 2), we observed positive changes in most BSID-III subscales Z-scores over the 6-month period in both

Confidential: For Review Only

224 study arms (median duration between measurements 189 days [IQR, 182 – 189]). Average
225 change for subscales was 0.45 (95% CI 0.23 – 0.67) Z-scores in the intervention arm, and 0.43
226 (95% CI 0.25 – 0.61) Z-scores in the usual care arm. No statistically significant differences were
227 observed between the two study arms.

228

**Z-scores Change for BSID-III Subscales[1]**	**Individualized Education (Intervention) Arm (n=47)**	**Usual Care Arm (n=53)**	**Difference**
Cognitive	0.28 [-0.14 – 0.71]	0.38 [0.05 – 0.72]	-0.10 [-0.63 – 0.43]
Receptive Language	0.49 [0.11 – 0.86]	0.56 [0.21 – 0.92]	-0.08 [-0.58 – 0.43]
Expressive Language	0.69 [0.38 – 0.99]	0.63 [0.34 – 0.93]	0.05 [-0.36 – 0.48]
Fine Motor	0.40 [0.04 – 0.76]	0.27 [-0.09 – 0.63]	0.13 [-0.38 – 0.63]
Gross Motor	0.70 [0.30 – 1.10]	0.51 [0.18 – 0.85]	0.19 [-0.32 – 0.70]
Socioemotional	0.20 [-0.13 – 0.53]	0.44 [0.07 – 0.81]	-0.24 [-0.73 – 0.25]

229 **Table 2: Key Developmental Outcomes**

230 [1] Values are average Z-score change [95% CI]

231

232

233 *Exploratory Analyses.* We used a hierarchical linear regression model to estimate changes
234 in each BSID-III subscale Z-score as a function of prespecified covariates, while controlling for
235 subject-level variation over time. Our final model included gender, age at enrollment, maternal
236 parity and school attendance, number of children under five in the household, and study arm. The
237 adjusted analysis was consistent with our unadjusted primary analysis, with statistically
238 significant improvements in developmental outcomes over the study period in all subscales
239 (Table 3), despite no difference between the study arms. Maternal school attendance was

Confidential: For Review Only

associated with greater positive expressive language and gross motor developmental changes (change at 6 months of 0.34, 95% CI 0.06 – 0.62, and 0.31, 95% CI 0.04 – 0.59 Z-scores respectively). Improvements in the cognitive subscale Z-scores were more pronounced for males (0.28, 95% CI 0.03 – 0.53).

Table 3. Estimates and 95% CI from Hierarchical Linear Mixed Models for Z-scores Change in BSID-III subscales

**Covariates**	**Cognitive** (n=176)	**Receptive Language** (n=176)	**Expressive Language** (n=174)	**Fine Motor** (n=174)	**Gross Motor** (n=175)	**Socio-emotional** (n=185)
Gender (0=female, 1=male)	0.28* (0.03,0.53)	-0.07 (-0.32,0.18)	-0.08 (-0.33,0.18)	0.03 (-0.21,0.26)	-0.05 (-0.30,0.21)	-0.06 (-0.32,0.19)
Age at enrollment (months)						
6 to 12	--	--	--	--	--	--
13 to 18	0.08 (-0.23,0.39)	0.06 (-0.24,0.37)	-0.13 (-0.45,0.18)	0.13 (-0.16,0.42)	0.07 (-0.24,0.38)	-0.09 (-0.41,0.24)
19 to 25	0.22 (-0.10,0.53)	0.07 (-0.24,0.38)	0.06 (-0.26,0.37)	0.20 (-0.10,0.50)	0.20 (-0.11,0.52)	-0.15 (-0.48,0.17)
Maternal parity (<=2, >=3)	-0.26 (-0.05,0.51)	-0.02 (-0.32,0.28)	-0.09 (-0.39,0.22)	-0.14 (-0.43,0.15)	-0.09 (-0.40,0.21)	-0.25 (-0.54,0.05)
Maternal school attendance (none, some)	-0.02 (-0.30,0.26)	0.10 (-0.17,0.38)	0.34* (0.06,0.62)	0.14 (-0.12,0.40)	0.31* (0.04,0.59)	-0.05 (-0.33,0.24)
#children under 5						
1	--	--	--	--	--	--
2	0.23 (-0.05,0.51)	0.02 (-0.26,0.30)	0.00 (-0.28,0.28)	0.14 (-0.12,0.41)	0.02 (-0.26,0.30)	-0.25 (-0.54,0.05)
>=3	0.36 (-0.10,0.82)	-0.13 (-0.59,0.32)	0.19 (-0.27,0.66)	0.08 (-0.36,0.51)	0.28 (-0.18,0.74)	-0.16 (-0.63,0.32)
Study time (0=entry, 1=exit)	0.41* (0.09,0.73)	0.50** (0.18,0.81)	0.69** (0.42,0.96)	0.32* (0.01,0.63)	0.58** (0.27,0.89)	0.52** (0.22,0.82)
Study arm (0=usual care, 1=intervention)	-0.08 (-0.38,0.21)	-0.04 (-0.32,0.25)	0.01 (-0.27,0.29)	-0.27 (-0.55,0.00)	-0.20 (-0.49,0.09)	-0.01 (-0.30,0.28)
LR test	0.11	0.13	0.0008	0.20	0.05	0.007

*p < 0.05, ** p <0.01

DISCUSSION

Confidential: For Review Only

In this paper, we describe developmental outcomes from a sub-study of a larger individually randomized clinical trial of a complementary feeding intervention in a rural indigenous area of Guatemala with high stunting prevalence. We found significant improvements across multiple developmental subscales over the study period for children in both the usual care (average change of 0.43 [95% CI 0.25 – 0.61] Z-scores) and the intervention (average change of 0.45 [95% CI 0.23 – 0.67] Z-scores) arms. These improvements remained highly statistically significant after controlling for important covariates (Table 3) and occurred despite the larger study showing only non-significant improvements in linear growth, as previously reported.[20]

No statistically significant difference in improvements were observed between the study arms, suggesting that both usual care and intensive approaches were equally effective in promoting development. Important limitations of our study lead to two alternative explanations for this finding. First, when designing the trial, usual care was to be delivered by an existing public sector rural program. However, widespread closures of these public services happened during study preparation.[35] Therefore, MHA's CHWs agreed to institute the usual care arm. Since these CHWs conduct activities using home visits (rather than the public sector's facility-based approach), the quality of usual care may have been greater than anticipated, leading to better outcomes. Additionally, the number of children included in the primary analysis was underpowered to detect a difference between the study arms. This loss of power occurred for two reasons. First an ethics board interim review request after completing data collection led to use study-internal baseline Z-scores for comparison and exclusion of some older children from the analysis. Second, the difficult rural geography and lengthy time-requirements for BSID-III assessments led to more caregivers than expected declining to complete follow-up assessments.

Confidential: For Review Only

Reassuringly, there were few differences in baseline characteristics between subjects included versus excluded for these two reasons (Supplementary Tables 1-2).

Our study also has two important strengths. First, despite extensive work on stunting in rural indigenous Maya populations, there have been few contemporary efforts to investigate developmental outcomes in this population.[8-11] Our study represents, to our knowledge, the first report of the impact of a nutrition intervention on development in Maya infants. Second, despite its impact on overall study power, the use of study-internal Z-scores for comparisons overcomes some concerns about the validity and reliability of the BSID-III for this indigenous population, allowing for robust internal comparisons.

Our study contributes to the literature on complementary feeding interventions, a cornerstone of stunting prevention and treatment efforts in low and middle-income countries.[13-15] Although multiple studies and meta-analyses have demonstrated the importance of complementary feeding interventions for promoting linear growth,[13-15] there are fewer studies examining their impact on development.[17, 18, 36] Here, we show that both usual care (lower intensity) and a higher-intensity approach to complementary feeding have important developmental effects. Our finding of no difference between higher- and lower-intensity approaches contrasts with a large cluster-randomized trial in India, where a more-intensive approach showed improved developmental outcomes.[37] Given our study's low power, and the fact that complementary feeding indicators significantly improved in our larger trial,[20] we believe that the final role of individualized, intensive approaches to complementary feeding in our setting is not yet settled.

Our study has two important implications for child nutrition policy in Guatemala. Guatemala's indigenous Maya population has some of the poorest nutritional outcomes in the

Confidential: For Review Only

world and, despite a recent resurgence public interest in this problem,[38, 39] chronic political and financial instability threatens sustained public and private commitments. Our study demonstrates, for the first time, the developmental impacts that such interventions can have for Maya children at risk and will help to advance this critical national conversation. Furthermore, our study shows that even low-intensity interventions modelled on existing public policy guidelines can be of great benefit. [40] Second, we demonstrate here that, despite the cultural and linguistic challenges of developmental assessments in this population, such evaluations are feasible and can show important developmental impact even when growth outcomes are equivocal.[20] We call for other nutrition researchers and program implementers in Guatemala to more routinely incorporate developmental outcomes into their planned evaluations.

Future research priorities for our group include larger-scale well-powered investigations of complementary and responsive feeding interventions of various intensity, as well as integrated nutrition and ECD interventions by CHWs in rural Guatemala. In addition, we are currently planning a reenrollment study of this trial cohort to see if further growth or developmental benefits emerge or are sustained with longer follow-up.

Confidential: For Review Only

TABLES/FIGURES:

Figure 1: Subject enrollment, randomization, and follow-up

Table 1: Baseline Demographic, Clinical and BSID-III Characteristics of Study Participants

Table 2: Key Developmental Outcomes

Table 3: Estimates and 95% CI from Hierarchical Linear Mixed Models for Z-scores Change in BSID-III subscales

Figure S1: Visual Likert scale for the BSID-III Socioemotional scales.

Table S1: Baseline Characteristics of Participants who completed the Study versus Lost to Follow-up or Unable to Complete BSID-III data Collection

Table S2: Baseline Characteristics of Study Participants Included versus Excluded in Outcome Analysis

Confidential: For Review Only

FIGURE LEGENDS

Figure 1: Subject enrollment, randomization, and follow-up.

Supplementary Figure 1: Likert scale containing oranges and baskets, used as visual aid for the BSID-III socioemotional scales data collection procedures. The left-most illustration –immature orange– represented the category 'Non-applicable' meaning that the child was too young to perform an assessment. The subsequent empty basket represented that the child 'never' performed an assessment but was already on the normal age-range to perform. The following incremental number of oranges within the baskets represented the categories that the child performed the assessment 'sometimes', 'half of the time', 'the majority of time', and 'always' respectively, according to the frequency that the child performed the assessment. The drawings were chosen and deemed culturally appropriate by caregivers and interpreters participating in BSID-III training procedures.

Confidential: For Review Only

LIST OF ABBREVIATIONS AND ACRONYMS

BSID-III: Bayley Scales of Infant Development, Version III

CHW: Community Health Worker

CI: Confidence Interval

ECD: Early Child Development

MHA: Maya Health Alliance

SD: Standard Deviations

WHO: World Health Organization

Confidential: For Review Only

FUNDING

This work was supported by Grand Challenges Canada, grant number SB-1726251050.

The study sponsor had no role in (1) study design; (2) the collection, analysis, and interpretation of data; (3) the writing of the report; and (4) the decision to submit the paper for publication.

Confidential: For Review Only

COMPETING INTERESTS

BM, MFW, and PR are employees or volunteers at Wuqu' Kawoq | Maya Health Alliance, the nongovernmental organization which provided logistical support for this study in Guatemala. SC, PR, ML, AG and MPG are doctoral students or employees of Universidad del Valle de Guatemala. The authors declare no other relationships or activities that could have influenced the submitted work.

Confidential: For Review Only

AUTHOR'S CONTRIBUTIONS

BM supervised the study, analyzed and interpreted data, and produced the first manuscript draft. SC cured, analyzed and interpreted data. PR, ML, AG collected data and supervised BSID-III data collection. MFW and MPG supervised the study and cultural adaptation for the BSID-III. MPG and PR designed and supervised the study. No individual was given any form of payment to produce the article. All authors critically revised the manuscript and made substantial contributions to the final draft.

Confidential: For Review Only

DATA SHARING STATEMENT

Replication dataset and statistical code are available upon publication at: https://doi.org/10.7910/DVN/ATJ3NW

Confidential: For Review Only

ACKNOWLEDGMENTS

We thank the subjects, their families, and communities for their participation in this study. We also thank the interpreters at MHA and the graduate and undergraduate psychology students at Universidad del Valle de Guatemala for their collaboration performing the BSID-III assessments.

Confidential: For Review Only

REFERENCES

1. Lu C, Black MM, Richter LM. Risk of poor development in young children in low-income and middle-income countries: an estimation and analysis at the global, regional, and country level. *Lancet Glob Health* 2016;4(12):e916–e22.

2. McCoy DC, Peet ED, Ezzati M, et al. Early Childhood Developmental Status in Low- and Middle-Income Countries: National, Regional, and Global Prevalence Estimates Using Predictive Modeling. *PLoS Med* 2016;13(6):e1002034.

3. Black RE, Victora CG, Walker SP, et al. Maternal and child undernutrition and overweight in low-income and middle-income countries. *Lancet* 2013;382(9890):427–51.

4. Ministerio de Salud Pública y Asistencia Social. VI Encuesta Nacional de Salud Materno Infantil. Guatemala City, Guatemala: Ministerio de Salud Pública y Asistencia Social; 2015.

5. Food and Nutritional Assistance III Project. Summary report: Development of evidence-based dietary recommendations for children, pregnant women, and lactating women living in the western highlands in Guatemala. Washington, DC: Food and Nutritional Assistance III Project; 2013.

6. Brown K, Henretty N, Chary A, et al. Mixed-methods study identifies key strategies for improving infant and young child feeding practices in a highly stunted rural indigenous population in Guatemala. *Matern Child Nutr* 2016;12(2):262–77.

7. Hoddinott J, Behrman JR, Maluccio JA, et al. Adult consequences of growth failure in early childhood. *Am J Clin Nutr* 2013;98(5):1170–8.

8. Varela-Silva MI, Bogin B, Sobral JA, et al. Deep data science to prevent and treat growth faltering in Maya children. *Eur J Clin Nutr* 2016;70(6):679–80.

Confidential: For Review Only

417 9. Kuklina EV, Ramakrishnan U, Stein AD, et al. Growth and diet quality are associated
418 with the attainment of walking in rural Guatemalan infants. *J Nutr* 2004;134(12):3296–300.
419 10. Thompson L, Penaloza RA, Stormfields K, et al. Validation and adaptation of rapid
420 neurodevelopmental assessment instrument for infants in Guatemala. *Child Care Health Dev*
421 2015;41(6):1131–9.
422 11. Kuklina EV, Ramakrishnan U, Stein AD, et al. Early childhood growth and development
423 in rural Guatemala. *Early Hum Dev* 2006;82(7):425–33.
424 12. Enneman A, Hernandez L, Campos R, et al. Dietary characteristics of complementary
425 foods offered to Guatemalan infants vary between urban and rural settings. *Nutr Res*
426 2009;29(7):470–9.
427 13. Lassi ZS, Das JK, Zahid G, et al. Impact of education and provision of complementary
428 feeding on growth and morbidity in children less than 2 years of age in developing countries: a
429 systematic review. *BMC Public Health* 2013;13 (Suppl 3):S13.
430 14. Dewey KG, Adu-Afarwuah S. Systematic review of the efficacy and effectiveness of
431 complementary feeding interventions in developing countries. *Matern Child Nutr* 2008;4 (Suppl
432 1):24–85.
433 15. Bhutta ZA, Das JK, Rizvi A, et al. Evidence-based interventions for improvement of
434 maternal and child nutrition: what can be done and at what cost? *Lancet* 2013;382(9890):452–77.
435 16. Krebs NF, Lozoff B, Georgieff MK. Neurodevelopment: The Impact of Nutrition and
436 Inflammation During Infancy in Low-Resource Settings. *Pediatrics* 2017;139(Suppl 1):S50–S8.
437 17. Walker SP, Chang SM, Powell CA, et al. Effects of early childhood psychosocial
438 stimulation and nutritional supplementation on cognition and education in growth-stunted
439 Jamaican children: prospective cohort study. *Lancet* 2005;366(9499):1804–7.

Confidential: For Review Only

18. Grantham-McGregor SM, Fernald LC, et al. Effects of integrated child development and nutrition interventions on child development and nutritional status. *Ann N Y Acad Sci* 2014;1308:11–32.

19. Martinez B, Flood D, Cnop K, et al. Improving Infant and Young Child Nutrition in a Highly Stunted Rural Community: A Practical Case Study from Guatemala. In: Preedy V, Patel V, eds. Handbook of Famine, Starvation, and Nutrient Deprivation. Cham, ZG: Springer. Published Online First: 27 June 2017. doi: 10.1007/978-3-319-40007-5_52-1

20. Martinez B, Webb MF, Gonzalez A, et al. Complementary feeding intervention on stunted Guatemalan children: a randomised controlled trial. *BMJ Paeds Open* 2018;2(1):e000213.

21. WHO Multicentre Growth Reference Study Group. WHO Child Growth Standards based on length/height, weight and age. *Acta Paediatr Suppl* 2006;450:76–85.

22. Hamadani JD, Tofail F, Hilaly A, et al. Use of family care indicators and their relationship with child development in Bangladesh. *J Health Popul Nut* 2010;1:23.

23. Bayley N. Bayley Scales of infant and toddler development. 3rd edn. San Antonio, TX: Harcourt Assessment 2006.

24. World Health Organization. Indicators for assessing infant and young child feeding practices. Washington, D.C.: World Health Organization; 2007.

25. Pollitt E, Saco-Pollitt C, Leibel RL, et al. Iron deficiency and behavioral development in infants and preschool children. *Am J Clin Nutr* 1986;43(4):555–65.

26. Fischer VJ, Morris J, Martines J. Developmental screening tools: feasibility of use at primary healthcare level in low-and middle-income settings. *J Health Popul Nutr* 2014;32(2):314.

Confidential: For Review Only

27. Sabanathan S, Wills B, Gladstone M. Child development assessment tools in low-income and middle-income countries: how can we use them more appropriately? *Arch Dis Child* 2015;100(5):482–8.

28. Bayley N. Escalas Bayley de desarrollo infantil - III. Madrid, ESP: Pearson Clinical 2015.

29. Schreiner M, Woller G. Simple Poverty Scorecard, Guatemala. 2010.

30. Cromwell EA, Dube Q, Cole SR, et al. Validity of US norms for the Bayley Scales of Infant Development-III in Malawian children. *Eur J Paediatr Neurol* 2014;18(2):223–30.

31. Chinta S, Walker K, Halliday R, et al. A comparison of the performance of healthy Australian 3-year-olds with the standardised norms of the Bayley Scales of Infant and Toddler Development (version-III). *Arch Dis Child* 2014;99(7):621–4.

32. Prado EL, Abbeddou S, Yakes Jimenez E, et al. Lipid-Based Nutrient Supplements Plus Malaria and Diarrhea Treatment Increase Infant Development Scores in a Cluster-Randomized Trial in Burkina Faso–3. *J Nutr* 2015;146(4):814–22.

33. Prado EL, Sebayang SK, Apriatni M, et al. Maternal multiple micronutrient supplementation and other biomedical and socioenvironmental influences on children's cognition at age 9–12 years in Indonesia: follow-up of the SUMMIT randomised trial. *Lancet Glob Health* 2017;5(2):e217–28.

34. West BT, Welch KB, Galecki AT. Linear mixed models: a practical guide using statistical software. Boca Raton, FL: CRC Press 2014:P440.

35. Avila C, Bright R, Gutierrez JC, et al. Guatemala health system assessment. Bethesda MD: Health Finance & Governance Project, Abt Associates Inc 2015.

Confidential: For Review Only

36. Larson LM, Yousafzai AK. A meta□analysis of nutrition interventions on mental development of children under□two in low□and middle□income countries. *Matern Child Nutr* 2017;13(1).

37. Vazir S, Engle P, Balakrishna N, et al. Cluster□randomized trial on complementary and responsive feeding education to caregivers found improved dietary intake, growth and development among rural Indian toddlers. *Matern Child Nutr* 2013;9(1):99–117.\

38. Government of Guatemala. Plan de Pacto Hambre Cero. Guatemala City, Guatemala; 2012.

39. Mejoremos Guate, Presentación lanzamiento alianza por la nutrición (Guatemala: Mejoremos Guate, 2012). Available at http://www.mejoremosguate.org/cms/content/files/que-estamos-haciendo/Presentacion_Lanzamiento_Alianza_por_la_Nutricion.pdf (Accessed 30 Apr 2018).

40. Gobierno de Guatemala, Secretaría de Planificación y Programación de la Presidencia. Lineamientos generales de política 2017-2019. Guatemala: Segeplán; 2017.

Confidential: For Review Only

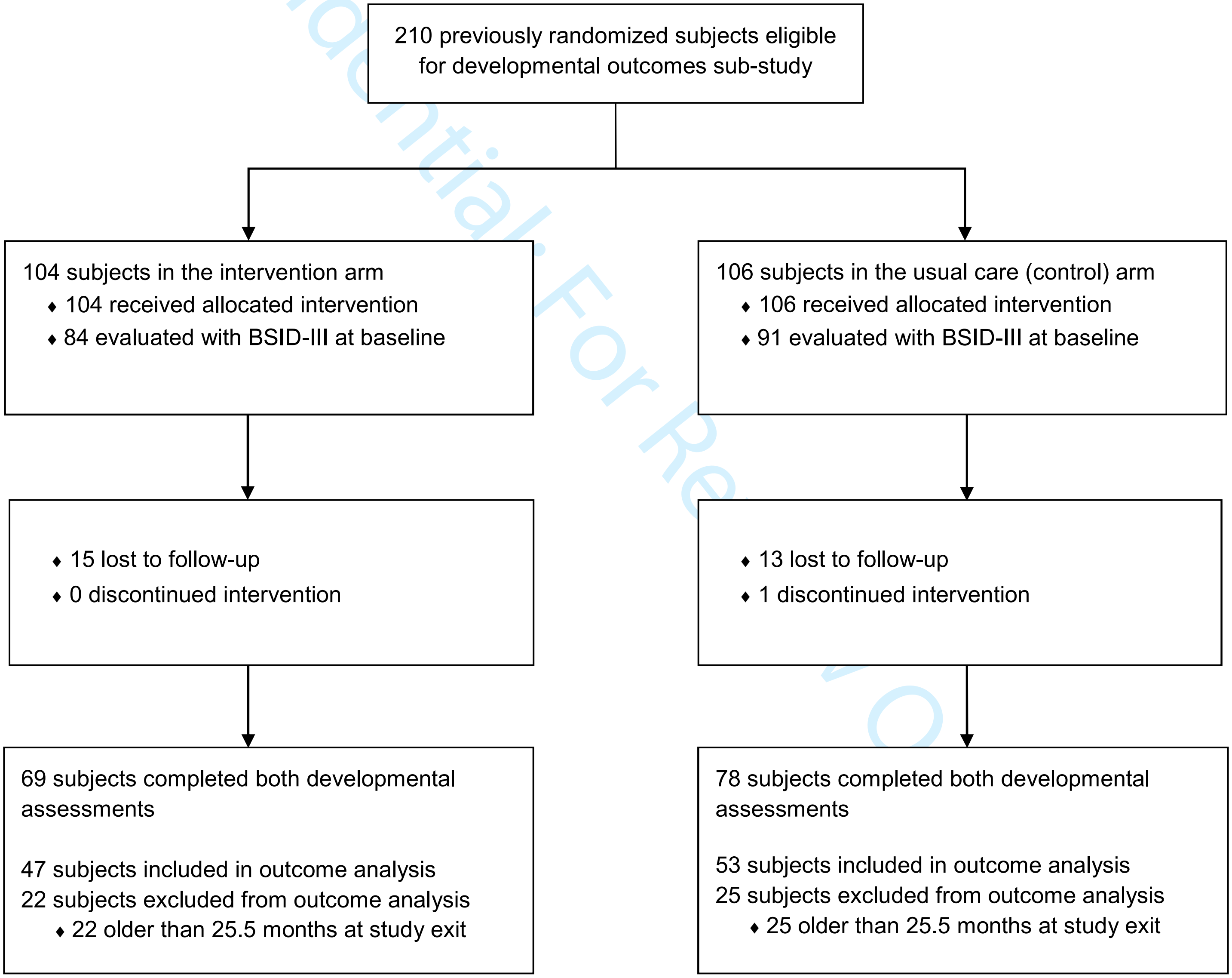
210 previously randomized subjects eligible for developmental outcomes sub-study
104 subjects in the intervention arm
104 received allocated intervention
84 evaluated with BSID-III at baseline
106 subjects in the usual care (control) arm
106 received allocated intervention
91 evaluated with BSID-III at baseline
15 lost to follow-up
0 discontinued intervention
13 lost to follow-up
1 discontinued intervention
69 subjects completed both developmental assessments
47 subjects included in outcome analysis
22 subjects excluded from outcome analysis
22 older than 25.5 months at study exit
78 subjects completed both developmental assessments
53 subjects included in outcome analysis
25 subjects excluded from outcome analysis
25 older than 25.5 months at study exit

Supplementary Table 1: Baseline Characteristics of Participants who Completed the Study vs Lost to Follow-up or Unable to Complete BSID-III Data Collection

Characteristics[1]	Completed Study (N=147)	Lost to follow up or Incomplete BSID-III (N=63)	P value[2]
**Maternal Characteristics**			
Age – yrs	27.07 ± 6.81	26.54 ± 6.41	0.60
Education – yrs	2 [0 – 4]	2 [0 – 3]	0.90
Literacy – no. (%)	72 (50)	34 (54)	0.51
Parity	3 [2 – 5]	3 [2 – 4]	0.62
**Child Characteristics**			
Male – no. (%)	89 (61)	37 (59)	0.81
Age at BSID III evaluation – months[3]	16.27 ± 5.18	15.00 ± 4.34	0.22
Height-for-age Z-score	-3.37 ± 0.66	-3.49 ± 0.87	0.31
Weight-for-age Z-score	-1.95 ± 0.77	-2.02 ± 0.79	0.52
Weight-for-length Z-score	-0.20 ± 0.91	-0.23 ± 0.76	0.80
**Feeding Practices Indicators**			
Minimum dietary diversity – no. (%)	88 (60)	42 (67)	0.35
Minimum meal frequency – no. (%)	122 (83)	58 (92)	0.13
Minimum acceptable diet – no. (%)	74 (50)	41 (62)	0.05
**Household Characteristics**			
Family Poverty Score	27.23 ± 10.08	27.65 ± 11.52	0.79
Family Care Indicators Score	8.54 ± 2.33	8.49 ± 2.46	0.90
**BSID-III Subscales Z-scores[3]**			
Cognitive	0.00 ± 1.00	-0.02 ± 0.89	0.93
Receptive Language	-0.01 ± 0.96	0.06 ± 1.12	0.73
Expressive Language	-0.01 ± 0.96	0.04 ± 1.09	0.83
Fine Motor	-0.01 ± 1.01	0.04 ± 0.83	0.81
Gross Motor	0.03 ± 0.98	-0.18 ± 0.98	0.31
Socioemotional	0.06 ± 1.05	-0.25 ± 1.03	0.15

[1] Plus minus values are means ± SD. [2]P values calculated using Student's t-test or Wilcoxon Mann-Whitney text for continuous variables, and the Chi2 or Fischer's exact test for categorical variables, as appropriate. [3] Participants with BSID-III at study entry who were lost to follow up are included in the third column (n=28).

Confidential: For Review Only

Supplementary Table 2: Baseline Characteristics of Participants Included versus Excluded in Outcome Analysis

Characteristics[1]	Included (N=100)	Excluded (N=47)	P value[2]
**Maternal Characteristics**			
Age – yrs	27.14 ± 6.61	26.94 ± 7.28	0.87
Education – yrs	2 [0 – 4]	2 [0 – 4]	0.20
Literacy – no. (%)	54 (54)	18 (38)	0.08
Parity	3 [2 – 5]	2 [1 – 5]	0.16
**Child Characteristics**			
Male – no. (%)	59 (59)	30 (64)	0.58
Age at BSID III evaluation – months	13.60 ± 3.95	21.97 ± 1.71	0.0000
Height-for-age Z-score	-3.33 ± 0.71	-3.47 ± 0.55	0.22
Weight-for-age Z-score	-1.91 ± 0.79	-2.04 ± 0.73	0.32
Weight-for-length Z-score	-0.10 ± 0.93	-0.41 ± 0.83	0.05
**Feeding Practices Indicators**			
Minimum dietary diversity – no. (%)	54 (54)	34 (72)	0.05
Minimum meal frequency – no. (%)	88 (88)	34 (72)	0.03
Minimum acceptable diet – no. (%)	49 (49)	25 (53)	0.64
**Household Characteristics**			
Family Poverty Score	27.12 ± 10.61	27.47 ± 8.95	0.85
Family Care Indicators Score	8.47 ± 2.41	8.68 ± 2.15	0.61

[1] Plus minus values are means ± SD. [2]P values calculated using Student's t-test or Wilcoxon Mann-Whitney text for continuous variables, and the Chi2 or Fischer's exact test for categorical variables, as appropriate.

Confidential: For Review Only

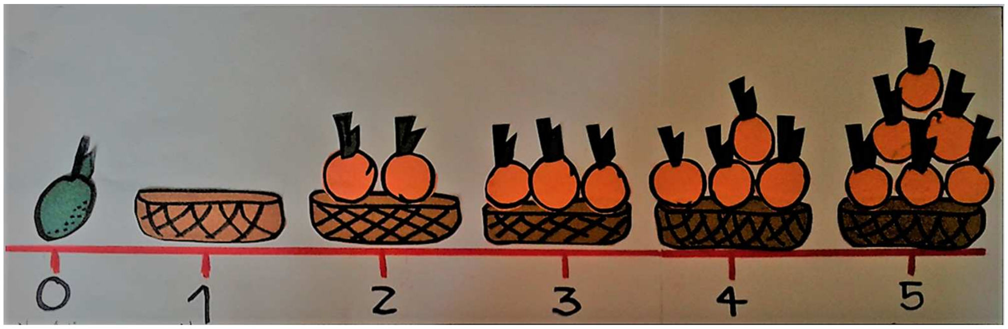

338x108mm (96 x 96 DPI)

Confidential: For Review Only

BMJ Paediatrics Open

Developmental outcomes of an individualized complementary feeding intervention for stunted children: A sub-study from a larger randomized controlled trial in Guatemala

Journal:	*BMJ Paediatrics Open*
Manuscript ID	bmjpo-2018-000314.R1
Article Type:	Original article
Date Submitted by the Author:	16-Jul-2018
Complete List of Authors:	Martinez, Boris; Wuqu' Kawoq,<br>Cardona, Sayra; Universidad del Valle de Guatemala<br>Rodas, Patricia; Universidad del Valle de Guatemala<br>Lubina, Meri; Universidad del Valle de Guatemala<br>Gonzalez, Ana; Universidad del Valle de Guatemala<br>Farley Webb, Meghan; Wuqu' Kawoq,<br>Grazioso, Maria del Pilar; Universidad del Valle de Guatemala<br>Rohloff, Peter
Keywords:	Child Psychology, General Paediatrics, Nutrition, Tropical Paediatrics

SCHOLARONE™
Manuscripts

Confidential: For Review Only

TITLE PAGE

Developmental outcomes of an individualized complementary feeding intervention for stunted children: A sub-study from a larger randomized controlled trial in Guatemala.

Boris Martinez MD[1,2], Sayra Cardona MA[3], Patricia Rodas MSc[3], Meri Lubina MA[3], Ana Gonzalez MA[3], Meghan Farley Webb PhD[1,4], Maria del Pilar Grazioso PhD[4], Peter Rohloff MD PhD[1,5,6]

[1]Wuqu' Kawoq | Maya Health Alliance, Santiago Sacatepéquez, Guatemala

[2]Department of Medicine, Saint Peter's University Hospital, New Brunswick, NJ, USA

[3]Department of Psychology, Universidad del Valle de Guatemala, Guatemala City, Guatemala

[4]Department of Anthropology & Sociology, Albion College, Albion, MI

[5]Division of Global Health Equity, Brigham and Women's Hospital, Boston, MA

[6]Corresponding Author. Peter Rohloff, Brigham and Women's Hospital, 75 Francis Street, Boston, MA, 02115. Phone: 617-278-0055. Fax: 888-372-2354. Email: prohloff@bwh.harvard.edu

Word count: 2538

Confidential: For Review Only

KEY WORDS:

Stunting, early child development, randomized clinical trial, Guatemala, indigenous, community health workers

Confidential: For Review Only

WHAT IS KNOWN ABOUT THIS SUBJECT

- Stunting is the most common cause of early childhood developmental delays, and nearly half of Guatemalan children are stunted, with higher prevalence among the Guatemalan Maya-indigenous population.
- Stunting early in life has deleterious long-term impacts on educational attainment, intellectual outcomes and adult economic earning potential. Few investigations of these effects exist for the Guatemalan indigenous Maya population.
- Complementary feeding practices interventions are the cornerstone for stunting prevention and treatment, however investigation of the effects of these interventions on developmental outcomes are limited.

WHAT THIS STUDY ADDS

- Both standard and augmented behavior change approaches to improved complementary feeding practices by caregivers have important positive impacts on the development of stunted children.
- Current public malnutrition reduction strategies being implemented in indigenous Maya communities in Guatemala can improve childhood developmental outcomes.
- Developmental assessments can be feasibly adapted and contextualized to a rural, indigenous population at risk.

Confidential: For Review Only

STRUCTURED ABSTRACT

Objective: Stunting is a common cause of early child developmental delay; Guatemala has the fourth highest rate of stunting globally. The goal of this study was to examine the impact of an intensive community health worker-led complementary feeding intervention on early child development in Guatemala. We hypothesized that the intervention would improve child development over usual care.

Design: A sub-study from a larger individually randomized (1:1 allocation ratio), parallel-group superiority trial, with blinding of study staff collecting outcomes data.

Setting: Rural, indigenous Maya communities in Guatemala.

Participants: 210 stunted children (height-for-age Z score less than or equal to -2.5) 6-24 months old, previously randomized to usual care (106) or an intensive complementary feeding intervention (104). 84 in the intervention and 91 in the usual care arm agreed to participate.

Interventions: Community health workers conducted monthly home visits for 6 months, providing usual care or individualized complementary feeding education.

Main Outcome Measures: The primary outcomes were change in Z-scores for the subscales of the Bayley Scales of Infant Development (BSID), Third Edition.

Results: 100 individuals were included in the final analysis, 47 in the intervention and 53 in the usual care arm. No statistically significant differences in age-adjusted scores between the arms were observed for any subscale. However, improvements within-subjects in both arms were observed (median duration between measurements 189 days [IQR 182 – 189]). Mean change for subscales was 0.45 (95% CI 0.23 – 0.67) Z-scores in the intervention, and 0.43 (95% CI 0.25 – 0.61) in the usual care arm.

Confidential: For Review Only

Conclusions: An intensive complementary feeding intervention did not significantly improve developmental outcomes more than usual care in stunted, indigenous Guatemalan children. However, both interventions had significant positive impacts on developmental outcomes.

Trial registration: Clinicaltrials.gov NCT02509936

Funding: Grand Challenges Canada grant SB-1726251050

Stage: Results

Confidential: For Review Only

INTRODUCTION

In low- and middle-income countries, 43% of children under five are at risk of not reaching their developmental potential, and 70% of the attributable risk for delays in early childhood development (ECD) is due to stunting, or low height-for-age.[1, 2] Guatemala, a Central American country of 17 million inhabitants, has the highest rate of stunting in the Western Hemisphere.[3] The burden of stunting disproportionately affects the rural, indigenous Maya population, where stunting often exceeds 70% and complementary feeding and dietary diversity indicators are poor.[4-6]

Early scientific research in Guatemala was critical to the international community's understanding of stunting's impact on human capital. The Institute of Nutrition of Central America and Panama cohort study—conducted from 1969-1977, with long-term follow-up—demonstrated the deleterious impacts of stunting on educational attainment, intellectual outcomes, and adult economic earning potential.[7] Subsequently, there have been extensive investigations of stunting's nutritional and sociodemographic correlates in the indigenous Maya population. However, there have been few investigations of developmental outcomes or of the impact of contemporary nutritional interventions on ECD.[8-11]

Given the gaps in adequate complementary feeding practices and diet quality, interventions to promote caregiver knowledge around infant-child feeding are an important strategy in Guatemala.[5, 6, 12] This strategy is supported by international evidence, with several studies showing that linear growth is strongly correlated with better development and that complementary feeding education interventions improve stunting.[11, 13-15] However, there has been limited direct investigation of the effect of complementary feeding on developmental outcomes.[16-18] This is an important knowledge gap, because feeding interventions may at

Confidential: For Review Only

times have small direct impacts on linear growth velocity but conceivably larger impact on developmental outcomes. Therefore, directly measuring these developmental outcomes may generate additional evidence supporting the interventions, which could have important policy implications in a country like Guatemala, where debate over the value of standard child nutrition program offerings is ongoing.[19,20]

In this study, we contribute by assessing developmental outcomes in indigenous Maya infants in rural Guatemala. We hypothesized that an individualized, intensive approach to caregiver complementary feeding education would improve outcomes over usual care. We individually randomized child-caregiver dyads (aged 6-24 months, height-for-age Z-score <-2.5) to 6 months of usual care, which included home-based growth monitoring and micronutrient and food supplementation, versus usual care augmented with individualized complementary feeding education, using monthly structured interviews and active goal-setting to promote incremental improvements.

METHODS

Study Context. This study was conducted at Maya Health Alliance (MHA), a primary healthcare organization working in rural Maya communities. At MHA, nutrition community health workers (CHW) provide home-based services to children aged 6-24 months with stunting.[20] The study was conducted in Tecpán, Chimaltenango (pop. 95,000), with a settlement of agricultural Kaqchikel Maya families living 25 kilometers from the town center. The study was conducted according to the principles of Declaration of Helsinki and approved by the Institutional Review Boards of the Universidad del Valle de Guatemala and Maya Health Alliance.

Confidential: For Review Only

Trial Design. This was a planned sub-study on developmental outcomes within a larger, individually randomized, two-arm trial comparing individualized complementary feeding caregiver education to usual care (Clinicaltrials.gov Identifier: NCT02509936), described in detail elsewhere.[20] Briefly, eligibility criteria included: children between 6-24 months-old and length-for-age Z score less than or equal to -2.5 standard deviations (SD) by World Health Organization (WHO) standards.[22] Exclusion criteria were acute malnutrition (weight-for-length Z score of <= -2 SD) or severe medical illness. Study interventions were delivered by two CHW teams, each consisting of 2 CHWs. One team provided usual care while the other provided the intervention. Child-caregiver dyads were recruited and written informed consent obtained by a study staff member not involved in the intervention. At enrollment, child's anthropometric and dietary feeding practices as well as household demographic and socioeconomic characteristics were obtained. Household developmental stimulation data was gathered at enrollment using the Family Care Indicators (FCI) interview.[23] Due to the considerable time and expense constraints related to administering psychometric testing, a subset of subjects, namely those consecutively recruited for the larger study during the first five months (n=210, Figure 1), were invited to participate in the sub-study. Study outcomes (subscale scores on the Bayley Scales of Infant Development, Third Edition [BSID-III]) [24] were obtained at 0 and 6 months by trained study psychologists from the Universidad del Valle de Guatemala, supported by MHA interpreters (Maya Kaqchikel/Spanish). Both psychologists and interpreters were blinded to study allocation.

Study Interventions. The study duration was 6 months. As described elsewhere,[21] the usual care arm received a monthly home-visit regimen from a team of CHWs including growth monitoring, daily multiple micronutrient powder supplement (ferrous fumarate 12.5 mg, zinc

Confidential: For Review Only

gluconate 5 mg, retinol acetate 300 mcg, folic acid 160 mcg, and ascorbic acid 30 mg; Prodipa S.A., Guatemala City, Guatemala); and a biweekly food ration (beans 1000 g., eggs 20 units, and *Incaparina* 900 g. [a common corn and soy-based food supplement; Alimentos S.A., Guatemala City, Guatemala]). For the intervention arm, subjects received usual care and monthly individualized caregiver counseling focused on improving meal frequency and dietary diversity for the child,[25] from a separate CHW team.

Study Outcomes and Measure. The primary outcome was a change in Z-score for subscales of the BSID-III, calculated as described below. In preparation for use of the BSID-III, our team conducted a review of prior studies of psychometric testing with Guatemalan children, with the majority using previous versions of the BSID.[9-11, 26] Despite its technical complexity, the BSID is widely-used as a gold-standard instrument worldwide.[27, 28] Additionally, our lead psychologist (MPG) had extensive prior experience successfully using the tool in Guatemala. For these reasons, the BSID-III was chosen.

In preparation for testing, our team (interpreters, linguists, psychologists, anthropologists, nurses and physicians) reviewed existing BSID-III English and Spanish materials.[24, 29] Modifications for idiomatic Guatemalan Spanish and translations into Kaqchikel Maya were produced through group discussion and audience testing with volunteer caregivers and health workers. Forward translation, back translation, harmonization and cognitive debriefing were used to ensure accuracy and cultural adaptation. A visual Likert scale assisted with administration of socioemotional scales (Supplementary Figure 1). A small number of vocabulary and visual stimulus items were substituted to be culturally or context appropriate (e.g., sedan car substituted for pick-up truck).

Confidential: For Review Only

167 Nine psychologists (bilingual Spanish/English; 3 postgraduate and 6 graduate or undergraduate) who had formal training in infant/child assessments as part of their education participated in a one week-long training in rural data collection and multicultural competencies, including active BSID-III practice sessions. Five Maya interpreters (Kaqchikel Maya/Spanish speaking; the majority with a high school teaching degree) participated in a one-week training with active BSID-III practice sessions. Senior study psychologist (MPG), with significant professional experience in infant/child assessments, supervised BSID-III training and data collection procedures. Testing was performed by a team of one psychologist and one interpreter, supervised by MPG and the principle study coordinator (BM).

176 *Sample Size and Randomization.* For the primary outcome, we calculated that enrollment of 72 subjects per arm would detect a change of 0.5 SD on the BSID-III composite score, with an alpha of 0.05, power of 80%, and 15% lost to follow-up. All subjects recruited for the larger study were allocated by simple randomization from a computer-generated list of random numbers, no further randomization or allocation were performed for this sub-study.

181 *Statistical Analyses.* Descriptive statistics were calculated using Stata 13. Family poverty scores (possible score range: 0-100) were calculated as described.[30] Overall FCI scores were calculated as the sum of 18 item scores.[23] Differences between arms were assessed using the Student's t-test or Wilcoxon-Mann-Whitney test for continuous variables and the chi-square or Fisher's exact test for categorical variables. Analysis was by intention-to-treat, except where loss to follow up prevented collecting outcome data or baseline age-band BSID-III data were unavailable to calculate exit BSID-III Z-scores.

188 Raw scores for each BSID-III subscale (cognitive, receptive and expressive language, fine and gross motor, socioemotional) were calculated as the sum of assessment items

Confidential: For Review Only

performed. BSID-III norms do not exist for Guatemala, and there are concerns about the validity of applying BSID-III norms from a different population. This is the case because, for example, some test items are most appropriate for urban, literate populations (e.g., use of books in the testing process) and because some language items require adaptation to the syntactic structure of Mayan languages.[31, 32] Furthermore, after data collection for the study was completed, interim ethics board review requested that we not use external norms in our analysis plan. Therefore study-internal Z-scores for each BSID-III subscale were calculated based on the raw scores distribution of each age-band during baseline measurements, an approach used in other studies.[33, 34] These were then used to calculate individual subject Z-scores for both 0 and 6 month timepoints. Since all subjects recruited were younger than 24 months-old, baseline BSID-III Z-score data distribution was unavailable for subjects in older age bands at study exit, hence subjects older than 24 months-old were excluded from the primary outcome analysis.

We conducted an adjusted exploratory analysis to investigate the effect of prespecified covariates on the primary outcomes, including maternal parity and education, gender, number of under-five children in the home, household socioeconomic status, and age and length-for-age at enrollment. We constructed a hierarchical linear model for BSID-III subscales (MIXED function, Stata 13). Non-significant covariates were removed from the model using serial likelihood ratio tests.[35] The best fit model for all BSID-III subscales was chosen using the Breusch-Pagan Langrange multiplier test.

RESULTS

Participants. Eligible participants were recruited in rolling fashion from August - December 2015. Final participants exited in July 2016. A total of 210 children were eligible for

https://mc.manuscriptcentral.com/bmjpo

the sub-study (104 intervention arm, 106 usual care arm). 84 children in the intervention and 91 in the control arm received BSID-III evaluation at study entry. Baseline characteristics of participants in the two study arms were well balanced, except for children in the intervention arm having greater minimum dietary diversity (Table 1).

Table 1: Baseline Demographic, Clinical, and BSID-III Characteristics of Study Participants

Characteristic[1]	Individualized Education (Intervention) Arm (N=84)	Usual Care Arm (N =91)	p Value[2]
**Maternal Characteristics**			
Age – yrs	26.08 ± 6.84	27.15 ± 6.59	0.29
Education – yrs	2 [0 – 3.5]	2 [0 – 4]	0.93
Literacy – no. (%)	44 (51.65)	47 (52.38)	0.92
Parity	2.5 [1 – 5]	3 [2 – 5]	0.43
**Child Characteristics**			
Male – no. (%)	49 (58.33)	57 (62.64)	0.56
Age at BSID III evaluation – months	16.32 ± 4.93	15.84 ± 5.21	0.53
Height-for-age Z-score	-3.40 ± 0.69	-3.41 ± 0.73	0.91
Weight-for-age Z-score	-2.01 ± 0.74	-1.94 ± 0.82	0.54
Weight-for-length Z-score	-0.26 ± 0.86	-0.13 ± 0.90	0.35
**Feeding Practices Indicators**			
Minimum dietary diversity – no. (%)	59 (70.24)	49 (53.85)	0.03
Minimum meal frequency – no. (%)	68 (80.95)	81 (89.01)	0.13
Minimum acceptable diet – no. (%)	51 (60.71)	43 (47.25)	0.07
**Household Characteristics**			
# Children under 5 years	2 [1 – 2]	2 [1 – 2]	0.18
Family Poverty Score	28.33 ± 11.64	27.35 ± 9.17	0.53
Family Care Indicators Score	8.59 ± 2.63	8.47 ± 2.15	0.74
**BSID-III Subscales Z-scores[3]**			
Cognitive	-0.05 ± 1.00	0.04 ± 0.97	0.53
Receptive Language	-0.02 ± 0.96	0.02 ± 1.00	0.81
Expressive Language	0.01 ± 0.94	-0.01 ± 1.02	0.88
Fine Motor	-0.15 ± 0.94	0.14 ± 1.00	0.06
Gross Motor	-0.10 ± 0.91	0.10 ± 1.04	0.18
Socioemotional	0.05 ± 1.15	-0.02 ± 0.95	0.65

[1] Data presented as means ± SD, median [IQR], or no. (%), as appropriate.

Confidential: For Review Only

[2] Students t-test or Wilcoxon-Mann-Whitney test for continuous variables, chi2 or Fischer's exact test for categorical variables, as appropriate.

The cumulative incidence of loss to follow-up was 18% (n=15) in the intervention and 14% (n=13) in the usual care arm (Figure 1). One child in the control arm discontinued treatment. Furthermore, 22 children in the intervention arm and 25 in the control arm with BSID-III data at study exit were older than 25.5 months-old. Baseline Z-score data distribution was not available for this older age-band. Supplementary Table 1 compares the characteristics of subjects who completed the study and both BSID-III evaluations (n=147) to those lost to follow-up or who did not complete both assessments (n=63). There were no statistically significant differences for major characteristics. Supplementary Table 2 compares the baseline characteristics of subjects excluded from the outcomes analysis due to age at study exit versus those included. Subjects excluded were similar to those included, except for having lower minimum meal frequency (72% for excluded vs. 88% for included subjects) and being older (21.97 ± 1.71 vs. 13.60 ± 3.95 months-old). Difference in age was an expected finding, since our analysis was restricted to subjects younger than 24 months at study endpoint, since baseline BSID-III data distribution for Z-scores calculation was unavailable for the older age bands.

Outcomes. The analysis of primary outcomes was by intention-to-treat for subjects younger than 24 months-old at study exit in this sub-study, including one child who discontinued treatment. 100 subjects were included in the primary analysis (47 intervention arm, 53 usual care arm; Figure 1). For developmental outcomes (Table 2), we observed positive changes in most BSID-III subscales Z-scores over the 6-month period in both study arms (median duration between measurements 189 days [IQR, 182 – 189]). Mean change for subscales was 0.45 (95% CI 0.23 – 0.67) Z-scores in the intervention arm, and 0.43 (95% CI 0.25 – 0.61) Z-scores in the

Confidential: For Review Only

usual care arm. No statistically significant differences were observed between the two study arms.

Z-scores Change for BSID-III Subscales[1]	Individualized Education (Intervention) Arm (n=47)	Usual Care Arm (n=53)	Difference
Cognitive	0.28 [-0.14 – 0.71]	0.38 [0.05 – 0.72]	-0.10 [-0.63 – 0.43]
Receptive Language	0.49 [0.11 – 0.86]	0.56 [0.21 – 0.92]	-0.08 [-0.58 – 0.43]
Expressive Language	0.69 [0.38 – 0.99]	0.63 [0.34 – 0.93]	0.05 [-0.36 – 0.48]
Fine Motor	0.40 [0.04 – 0.76]	0.27 [-0.09 – 0.63]	0.13 [-0.38 – 0.63]
Gross Motor	0.70 [0.30 – 1.10]	0.51 [0.18 – 0.85]	0.19 [-0.32 – 0.70]
Socioemotional	0.20 [-0.13 – 0.53]	0.44 [0.07 – 0.81]	-0.24 [-0.73 – 0.25]
**Mean Change**	**0.45 [0.23 – 0.67]**	**0.43 [0.25 – 0.61]**	**0.02 [-0.25 – 0.30]**

Table 2: Key Developmental Outcomes

[1] Values are mean Z-score change [95% CI]

Exploratory Analyses. We used a hierarchical linear regression model to estimate changes in each BSID-III subscale Z-score as a function of prespecified covariates, while controlling for subject-level variation over time. Our final model included gender, age at enrollment, maternal parity and school attendance, number of children under five in the household, and study arm. The adjusted analysis was consistent with our unadjusted primary analysis, with statistically significant improvements in developmental outcomes over the study period in all subscales (Table 3), despite no difference between the study arms. Maternal school attendance was associated with greater positive expressive language and gross motor developmental changes

Confidential: For Review Only

(change at 6 months of 0.34, 95% CI 0.06 – 0.62, and 0.31, 95% CI 0.04 – 0.59 Z-scores respectively). Improvements in the cognitive subscale Z-scores were more pronounced for males (0.28, 95% CI 0.03 – 0.53).

Table 3. Estimates and 95% CI from Hierarchical Linear Mixed Models for Z-scores Change in BSID-III subscales

**Covariates**	**Cognitive** (n=176)	**Receptive Language** (n=176)	**Expressive Language** (n=174)	**Fine Motor** (n=174)	**Gross Motor** (n=175)	**Socio-emotional** (n=185)
Adjusted Z-score change from study entry to exit	0.41* (0.09,0.73)	0.50** (0.18,0.81)	0.69** (0.42,0.96)	0.32* (0.01,0.63)	0.58** (0.27,0.89)	0.52** (0.22,0.82)
Gender (0=female, 1=male)	0.28* (0.03,0.53)	-0.07 (-0.32,0.18)	-0.08 (-0.33,0.18)	0.03 (-0.21,0.26)	-0.05 (-0.30,0.21)	-0.06 (-0.32,0.19)
Age at enrollment (months)						
6 to 12	--	--	--	--	--	--
13 to 18	0.08 (-0.23,0.39)	0.06 (-0.24,0.37)	-0.13 (-0.45,0.18)	0.13 (-0.16,0.42)	0.07 (-0.24,0.38)	-0.09 (-0.41,0.24)
19 to 24	0.22 (-0.10,0.53)	0.07 (-0.24,0.38)	0.06 (-0.26,0.37)	0.20 (-0.10,0.50)	0.20 (-0.11,0.52)	-0.15 (-0.48,0.17)
Maternal parity (<=2, >=3)	-0.26 (-0.05,0.51)	-0.02 (-0.32,0.28)	-0.09 (-0.39,0.22)	-0.14 (-0.43,0.15)	-0.09 (-0.40,0.21)	-0.25 (-0.54,0.05)
Maternal school attendance (none, some)	-0.02 (-0.30,0.26)	0.10 (-0.17,0.38)	0.34* (0.06,0.62)	0.14 (-0.12,0.40)	0.31* (0.04,0.59)	-0.05 (-0.33,0.24)
#children under 5						
1	--	--	--	--	--	--
2	0.23 (-0.05,0.51)	0.02 (-0.26,0.30)	0.00 (-0.28,0.28)	0.14 (-0.12,0.41)	0.02 (-0.26,0.30)	-0.25 (-0.54,0.05)
>=3	0.36 (-0.10,0.82)	-0.13 (-0.59,0.32)	0.19 (-0.27,0.66)	0.08 (-0.36,0.51)	0.28 (-0.18,0.74)	-0.16 (-0.63,0.32)
Study arm (0=usual care, 1=intervention)	-0.08 (-0.38,0.21)	-0.04 (-0.32,0.25)	0.01 (-0.27,0.29)	-0.27 (-0.55,0.00)	-0.20 (-0.49,0.09)	-0.01 (-0.30,0.28)
LR test	0.11	0.13	0.0008	0.20	0.05	0.007

*p < 0.05, ** p <0.01

DISCUSSION

Confidential: For Review Only

In this paper, we describe developmental outcomes from a sub-study of a larger individually randomized clinical trial of a complementary feeding intervention in a rural indigenous area of Guatemala with high stunting prevalence. We found significant improvements across multiple developmental subscales over the study period for children in both the usual care (mean change of 0.43 [95% CI 0.25 – 0.61] Z-scores) and the intervention (mean change of 0.45 [95% CI 0.23 – 0.67] Z-scores) arms. These improvements remained highly statistically significant after controlling for important covariates (Table 3) and occurred despite the larger study showing only non-significant improvements in linear growth, as previously reported.[21]

No statistically significant difference in improvements were observed between the study arms, suggesting that both usual care and intensive approaches were equally effective in promoting development. Important limitations of our study lead to two alternative explanations for this finding. First, when designing the trial, usual care was to be delivered by an existing public sector rural program. However, widespread closures of these public services happened during study preparation.[19] Therefore, MHA's CHWs agreed to institute the usual care arm. Since these CHWs conduct activities using home visits (rather than the public sector's facility-based approach), the quality of usual care may have been greater than anticipated, leading to better outcomes. Additionally, the number of children included in the primary analysis was underpowered to detect a difference between the study arms. This loss of power occurred for two reasons. First an ethics board interim review request after completing data collection led to use study-internal baseline Z-scores for comparison and exclusion of some older children from the analysis. Second, the difficult rural geography and lengthy time-requirements for BSID-III assessments led to more caregivers than expected declining to complete follow-up assessments. Although there were few differences in baseline characteristics between subjects included versus

excluded for these two reasons (Supplementary Tables 1-2), the proportion of children included in the analysis still remains only 43% of the originally randomized sample (Figure 1). This potentially limits the generalizability of our findings, especially for the older children (aged 25-30 months at study exit) who represent the largest proportion of subjects excluded from the analysis.

Our study also has two important strengths. First, despite extensive work on stunting in rural indigenous Maya populations, there have been few contemporary efforts to investigate developmental outcomes in this population.[8-11] Our study represents, to our knowledge, the first report of the impact of a nutrition intervention on development in Maya infants. Second, despite its impact on overall study power, the use of study-internal Z-scores for comparisons overcomes some concerns about the validity and reliability of the BSID-III for this indigenous population, allowing for robust internal comparisons, although the results we report here cannot be directly compared with populations from other studies..

Our study contributes to the literature on complementary feeding interventions, a cornerstone of stunting prevention and treatment efforts in low and middle-income countries.[13-15] Although multiple studies and meta-analyses have demonstrated the importance of complementary feeding interventions for promoting linear growth,[13-15] there are fewer studies examining their impact on development.[17, 18, 36] Here, we show that both usual care (lower intensity) and a higher-intensity approach to complementary feeding have important developmental effects. Our finding of no difference between higher- and lower-intensity approaches contrasts with a large cluster-randomized trial in India, where a more-intensive approach showed improved developmental outcomes.[37] Given our study's low power, and the fact that complementary feeding indicators significantly improved in our larger trial,[21] we

Confidential: For Review Only

believe that the final role of individualized, intensive approaches to complementary feeding in our setting is not yet settled.

Our study has two important implications for child nutrition policy in Guatemala. Guatemala's indigenous Maya population has some of the poorest nutritional outcomes in the world and, despite a recent resurgence public interest in this problem,[38, 39] chronic political and financial instability threatens sustained public and private commitments. Our study demonstrates, for the first time, the developmental impacts that such interventions can have for Maya children at risk and will help to advance this critical national conversation. Furthermore, our study shows that even low-intensity interventions modelled on existing public policy guidelines can be of great benefit.[40] Second, we demonstrate here that, despite the cultural and linguistic challenges of developmental assessments in this population, such evaluations are feasible and can show important developmental impact even when growth outcomes are equivocal.[21] We call for other nutrition researchers and program implementers in Guatemala to more routinely incorporate developmental outcomes into their planned evaluations.

Future research priorities for our group include larger-scale well-powered investigations of complementary and responsive feeding interventions, as well as more comprehensive, integrated nutrition and ECD interventions by CHWs in rural Guatemala. This latter point is especially important, since it is increasingly recognized that comprehensive, multisectorial interventions are most likely to generate sustained positive impact. Design and evaluation of comprehensive wrap-around interventions is also most in-line with the Nurturing Care Framework for childhood recently put forth by the WHO and UNICEF.[41,42] In addition, we are currently planning a reenrollment study of this trial cohort to see if further growth or developmental benefits emerge or are sustained with longer follow-up, since an inherent

Confidential: For Review Only

weakness of this study was its short follow-up time. Finally, we plan to publish in greater detail our methodology for adapting the BSID-III and related instruments to this population.

Confidential: For Review Only

TABLES/FIGURES:

Figure 1: Subject enrollment, randomization, and follow-up

Table 1: Baseline Demographic, Clinical and BSID-III Characteristics of Study Participants

Table 2: Key Developmental Outcomes

Table 3: Estimates and 95% CI from Hierarchical Linear Mixed Models for Z-scores Change in BSID-III subscales

Figure S1: Visual Likert scale for the BSID-III Socioemotional scales.

Table S1: Baseline Characteristics of Participants who completed the Study versus Lost to Follow-up or Unable to Complete BSID-III data Collection

Table S2: Baseline Characteristics of Study Participants Included versus Excluded in Outcome Analysis

Confidential: For Review Only

FIGURE LEGENDS

Figure 1: Subject enrollment, randomization, and follow-up.

Supplementary Figure 1: Likert scale containing oranges and baskets, used as visual aid for the BSID-III socioemotional scales data collection procedures. The left-most illustration –immature orange– represented the category ‘Non-applicable’ meaning that the child was too young to perform an assessment. The subsequent empty basket represented that the child ‘never’ performed an assessment but was already on the normal age-range to perform. The following incremental number of oranges within the baskets represented the categories that the child performed the assessment ‘sometimes’, ‘half of the time’, ‘the majority of time’, and ‘always’ respectively, according to the frequency that the child performed the assessment. The drawings were chosen and deemed culturally appropriate by caregivers and interpreters participating in BSID-III training procedures.

Confidential: For Review Only

LIST OF ABBREVIATIONS AND ACRONYMS

BSID-III: Bayley Scales of Infant Development, Version III

CHW: Community Health Worker

CI: Confidence Interval

ECD: Early Child Development

MHA: Maya Health Alliance

SD: Standard Deviations

WHO: World Health Organization

Confidential: For Review Only

FUNDING

This work was supported by Grand Challenges Canada, grant number SB-1726251050. The study sponsor had no role in (1) study design; (2) the collection, analysis, and interpretation of data; (3) the writing of the report; and (4) the decision to submit the paper for publication.

Confidential: For Review Only

COMPETING INTERESTS

BM, MFW, and PR are employees or volunteers at Wuqu' Kawoq | Maya Health Alliance, the nongovernmental organization which provided logistical support for this study in Guatemala. SC, PR, ML, AG and MPG are doctoral students or employees of Universidad del Valle de Guatemala. The authors declare no other relationships or activities that could have influenced the submitted work.

Confidential: For Review Only

AUTHOR'S CONTRIBUTIONS

BM supervised the study, analyzed and interpreted data, and produced the first manuscript draft. SC cured, analyzed and interpreted data. PR, ML, AG collected data and supervised BSID-III data collection. MFW and MPG supervised the study and cultural adaptation for the BSID-III. MPG and PR designed and supervised the study. No individual was given any form of payment to produce the article. All authors critically revised the manuscript and made substantial contributions to the final draft.

Confidential: For Review Only

412 **DATA SHARING STATEMENT**

Study protocol, replication dataset and statistical code are available upon publication at: https://doi.org/10.7910/DVN/ATJ3NW

Confidential: For Review Only

ACKNOWLEDGMENTS

We thank the subjects, their families, and communities for their participation in this study. We also thank the interpreters at MHA and the graduate and undergraduate psychology students at Universidad del Valle de Guatemala for their collaboration performing the BSID-III assessments.

Confidential: For Review Only

REFERENCES

1. Lu C, Black MM, Richter LM. Risk of poor development in young children in low-income and middle-income countries: an estimation and analysis at the global, regional, and country level. *Lancet Glob Health* 2016;4(12):e916–e22.

2. McCoy DC, Peet ED, Ezzati M, et al. Early Childhood Developmental Status in Low- and Middle-Income Countries: National, Regional, and Global Prevalence Estimates Using Predictive Modeling. *PLoS Med* 2016;13(6):e1002034.

3. Black RE, Victora CG, Walker SP, et al. Maternal and child undernutrition and overweight in low-income and middle-income countries. *Lancet* 2013;382(9890):427–51.

4. Ministerio de Salud Pública y Asistencia Social. VI Encuesta Nacional de Salud Materno Infantil. Guatemala City, Guatemala: Ministerio de Salud Pública y Asistencia Social; 2015.

5. Food and Nutritional Assistance III Project. Summary report: Development of evidence-based dietary recommendations for children, pregnant women, and lactating women living in the western highlands in Guatemala. Washington, DC: Food and Nutritional Assistance III Project; 2013.

6. Brown K, Henretty N, Chary A, et al. Mixed-methods study identifies key strategies for improving infant and young child feeding practices in a highly stunted rural indigenous population in Guatemala. *Matern Child Nutr* 2016;12(2):262–77.

7. Hoddinott J, Behrman JR, Maluccio JA, et al. Adult consequences of growth failure in early childhood. *Am J Clin Nutr* 2013;98(5):1170–8.

8. Varela-Silva MI, Bogin B, Sobral JA, et al. Deep data science to prevent and treat growth faltering in Maya children. *Eur J Clin Nutr* 2016;70(6):679–80.

Confidential: For Review Only

9. Kuklina EV, Ramakrishnan U, Stein AD, et al. Growth and diet quality are associated with the attainment of walking in rural Guatemalan infants. *J Nutr* 2004;134(12):3296–300.

10. Thompson L, Penaloza RA, Stormfields K, et al. Validation and adaptation of rapid neurodevelopmental assessment instrument for infants in Guatemala. *Child Care Health Dev* 2015;41(6):1131–9.

11. Kuklina EV, Ramakrishnan U, Stein AD, et al. Early childhood growth and development in rural Guatemala. *Early Hum Dev* 2006;82(7):425–33.

12. Enneman A, Hernandez L, Campos R, et al. Dietary characteristics of complementary foods offered to Guatemalan infants vary between urban and rural settings. *Nutr Res* 2009;29(7):470–9.

13. Lassi ZS, Das JK, Zahid G, et al. Impact of education and provision of complementary feeding on growth and morbidity in children less than 2 years of age in developing countries: a systematic review. *BMC Public Health* 2013;13 (Suppl 3):S13.

14. Dewey KG, Adu-Afarwuah S. Systematic review of the efficacy and effectiveness of complementary feeding interventions in developing countries. *Matern Child Nutr* 2008;4 (Suppl 1):24–85.

15. Bhutta ZA, Das JK, Rizvi A, et al. Evidence-based interventions for improvement of maternal and child nutrition: what can be done and at what cost? *Lancet* 2013;382(9890):452–77.

16. Krebs NF, Lozoff B, Georgieff MK. Neurodevelopment: The Impact of Nutrition and Inflammation During Infancy in Low-Resource Settings. *Pediatrics* 2017;139(Suppl 1):S50–S8.

17. Walker SP, Chang SM, Powell CA, et al. Effects of early childhood psychosocial stimulation and nutritional supplementation on cognition and education in growth-stunted Jamaican children: prospective cohort study. *Lancet* 2005;366(9499):1804–7.

Confidential: For Review Only

18. Grantham-McGregor SM, Fernald LC, et al. Effects of integrated child development and nutrition interventions on child development and nutritional status. *Ann N Y Acad Sci* 2014;1308:11–32.

19. Avila C, Bright R, Gutierrez JC, et al. Guatemala health system assessment. Bethesda MD: Health Finance & Governance Project, Abt Associates Inc 2015.

20. Martinez B, Flood D, Cnop K, et al. Improving Infant and Young Child Nutrition in a Highly Stunted Rural Community: A Practical Case Study from Guatemala. In: Preedy V, Patel V, eds. Handbook of Famine, Starvation, and Nutrient Deprivation. Cham, ZG: Springer. Published Online First: 27 June 2017. doi: 10.1007/978-3-319-40007-5_52-1

21. Martinez B, Webb MF, Gonzalez A, et al. Complementary feeding intervention on stunted Guatemalan children: a randomised controlled trial. *BMJ Paeds Open* 2018;2(1):e000213.

22. WHO Multicentre Growth Reference Study Group. WHO Child Growth Standards based on length/height, weight and age. *Acta Paediatr Suppl* 2006;450:76–85.

23. Hamadani JD, Tofail F, Hilaly A, et al. Use of family care indicators and their relationship with child development in Bangladesh. *J Health Popul Nut* 2010;1:23.

24. Bayley N. Bayley Scales of infant and toddler development. 3rd edn. San Antonio, TX: Harcourt Assessment 2006.

25. World Health Organization. Indicators for assessing infant and young child feeding practices. Washington, D.C.: World Health Organization; 2007.

26. Pollitt E, Saco-Pollitt C, Leibel RL, et al. Iron deficiency and behavioral development in infants and preschool children. *Am J Clin Nutr* 1986;43(4):555–65.

Confidential: For Review Only

27. Fischer VJ, Morris J, Martines J. Developmental screening tools: feasibility of use at primary healthcare level in low-and middle-income settings. *J Health Popul Nutr* 2014;32(2):314.

28. Sabanathan S, Wills B, Gladstone M. Child development assessment tools in low-income and middle-income countries: how can we use them more appropriately? *Arch Dis Child* 2015;100(5):482–8.

29. Bayley N. Escalas Bayley de desarrollo infantil - III. Madrid, ESP: Pearson Clinical 2015.

30. Schreiner M, Woller G. Simple Poverty Scorecard, Guatemala. 2010.

31. Cromwell EA, Dube Q, Cole SR, et al. Validity of US norms for the Bayley Scales of Infant Development-III in Malawian children. *Eur J Paediatr Neurol* 2014;18(2):223–30.

32. Chinta S, Walker K, Halliday R, et al. A comparison of the performance of healthy Australian 3-year-olds with the standardised norms of the Bayley Scales of Infant and Toddler Development (version-III). *Arch Dis Child* 2014;99(7):621–4.

33. Prado EL, Abbeddou S, Yakes Jimenez E, et al. Lipid-Based Nutrient Supplements Plus Malaria and Diarrhea Treatment Increase Infant Development Scores in a Cluster-Randomized Trial in Burkina Faso–3. *J Nutr* 2015;146(4):814–22.

34. Prado EL, Sebayang SK, Apriatni M, et al. Maternal multiple micronutrient supplementation and other biomedical and socioenvironmental influences on children's cognition at age 9–12 years in Indonesia: follow-up of the SUMMIT randomised trial. *Lancet Glob Health* 2017;5(2):e217–28.

35. West BT, Welch KB, Galecki AT. Linear mixed models: a practical guide using statistical software. Boca Raton, FL: CRC Press 2014:P440.

Confidential: For Review Only

36. Larson LM, Yousafzai AK. A meta□analysis of nutrition interventions on mental development of children under□two in low□and middle□income countries. *Matern Child Nutr* 2017;13(1).

37. Vazir S, Engle P, Balakrishna N, et al. Cluster□randomized trial on complementary and responsive feeding education to caregivers found improved dietary intake, growth and development among rural Indian toddlers. *Matern Child Nutr* 2013;9(1):99–117.\

38. Government of Guatemala. Plan de Pacto Hambre Cero. Guatemala City, Guatemala; 2012.

39. Mejoremos Guate, Presentación lanzamiento alianza por la nutrición (Guatemala: Mejoremos Guate, 2012). Available at http://www.mejoremosguate.org/cms/content/files/que-estamos-haciendo/Presentacion_Lanzamiento_Alianza_por_la_Nutricion.pdf (Accessed 30 Apr 2018).

40. Gobierno de Guatemala, Secretaría de Planificación y Programación de la Presidencia. Lineamientos generales de política 2017-2019. Guatemala: Segeplán; 2017.

41. Hossain M, Choudhury N, Adib Binte Abdullah K, et al. Evidence-based approaches to childhood stunting in low and middle income countries: a systematic review. *Arch Dis Child*. 2017;102(10):903-9.

42. World Health Organization, United Nations Children's Fund, World Bank Group. Nurturing care for early childhood development: a framework for helping children survive and thrive to transform health and human potential. Geneva: World Health Organization; 2018.

Confidential: For Review Only

Confidential: For Review Only

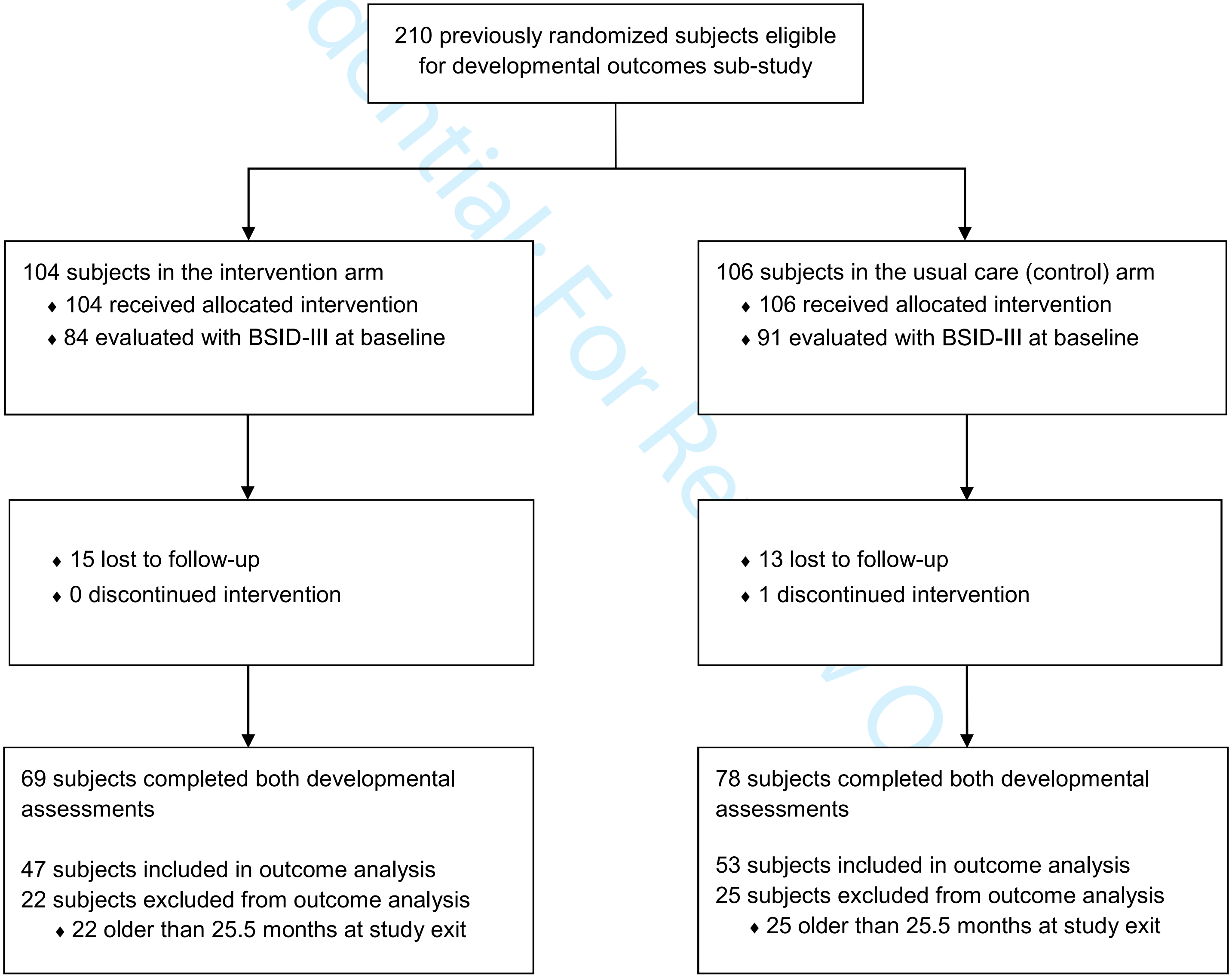

Confidential: For Review Only

Supplementary Table 1: Baseline Characteristics of Participants who Completed the Study vs Lost to Follow-up or Unable to Complete BSID-III Data Collection

Characteristics[1]	Completed Study (N=147)	Lost to follow up or Incomplete BSID-III (N=63)	P value[2]
**Maternal Characteristics**			
Age – yrs	27.07 ± 6.81	26.54 ± 6.41	0.60
Education – yrs	2 [0 – 4]	2 [0 – 3]	0.90
Literacy – no. (%)	72 (50)	34 (54)	0.51
Parity	3 [2 – 5]	3 [2 – 4]	0.62
**Child Characteristics**			
Male – no. (%)	89 (61)	37 (59)	0.81
Age at BSID III evaluation – months[3]	16.27 ± 5.18	15.00 ± 4.34	0.22
Height-for-age Z-score	-3.37 ± 0.66	-3.49 ± 0.87	0.31
Weight-for-age Z-score	-1.95 ± 0.77	-2.02 ± 0.79	0.52
Weight-for-length Z-score	-0.20 ± 0.91	-0.23 ± 0.76	0.80
**Feeding Practices Indicators**			
Minimum dietary diversity – no. (%)	88 (60)	42 (67)	0.35
Minimum meal frequency – no. (%)	122 (83)	58 (92)	0.13
Minimum acceptable diet – no. (%)	74 (50)	41 (62)	0.05
**Household Characteristics**			
Family Poverty Score	27.23 ± 10.08	27.65 ± 11.52	0.79
Family Care Indicators Score	8.54 ± 2.33	8.49 ± 2.46	0.90
**BSID-III Subscales Z-scores[3]**			
Cognitive	0.00 ± 1.00	-0.02 ± 0.89	0.93
Receptive Language	-0.01 ± 0.96	0.06 ± 1.12	0.73
Expressive Language	-0.01 ± 0.96	0.04 ± 1.09	0.83
Fine Motor	-0.01 ± 1.01	0.04 ± 0.83	0.81
Gross Motor	0.03 ± 0.98	-0.18 ± 0.98	0.31
Socioemotional	0.06 ± 1.05	-0.25 ± 1.03	0.15

[1] Plus minus values are means ± SD. [2]P values calculated using Student's t-test or Wilcoxon Mann-Whitney text for continuous variables, and the Chi2 or Fischer's exact test for categorical variables, as appropriate. [3] Participants with BSID-III at study entry who were lost to follow up are included in the third column (n=28).

Confidential: For Review Only

Supplementary Table 2: Baseline Characteristics of Participants Included versus Excluded in Outcome Analysis

Characteristics[1]	Included (N=100)	Excluded (N=47)	P value[2]
**Maternal Characteristics**			
Age – yrs	27.14 ± 6.61	26.94 ± 7.28	0.87
Education – yrs	2 [0 – 4]	2 [0 – 4]	0.20
Literacy – no. (%)	54 (54)	18 (38)	0.08
Parity	3 [2 – 5]	2 [1 – 5]	0.16
**Child Characteristics**			
Male – no. (%)	59 (59)	30 (64)	0.58
Age at BSID III evaluation – months	13.60 ± 3.95	21.97 ± 1.71	0.0000
Height-for-age Z-score	-3.33 ± 0.71	-3.47 ± 0.55	0.22
Weight-for-age Z-score	-1.91 ± 0.79	-2.04 ± 0.73	0.32
Weight-for-length Z-score	-0.10 ± 0.93	-0.41 ± 0.83	0.05
**Feeding Practices Indicators**			
Minimum dietary diversity – no. (%)	54 (54)	34 (72)	0.05
Minimum meal frequency – no. (%)	88 (88)	34 (72)	0.03
Minimum acceptable diet – no. (%)	49 (49)	25 (53)	0.64
**Household Characteristics**			
Family Poverty Score	27.12 ± 10.61	27.47 ± 8.95	0.85
Family Care Indicators Score	8.47 ± 2.41	8.68 ± 2.15	0.61

[1] Plus minus values are means ± SD. [2]P values calculated using Student's t-test or Wilcoxon Mann-Whitney text for continuous variables, and the Chi2 or Fischer's exact test for categorical variables, as appropriate.

Confidential: For Review Only

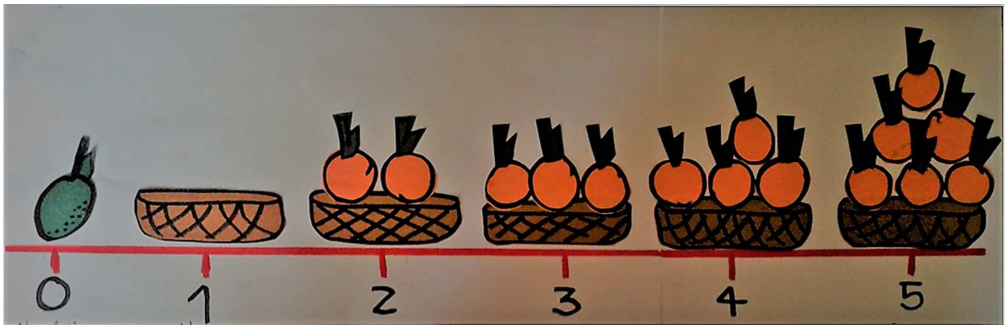

338x108mm (96 x 96 DPI)

Confidential: For Review Only
